# Exosomal LBH inhibits epithelial-mesenchymal transition and angiogenesis in nasopharyngeal carcinoma via downregulating VEGFA signaling

**DOI:** 10.7150/ijbs.66506

**Published:** 2022-01-01

**Authors:** Anbiao Wu, Ning Luo, Yuling Xu, Nan Du, Li Li, Qicai Liu

**Affiliations:** 1 Department of Cardiology, Laboratory of Heart Center; Guangdong Provincial Biomedical Engineering Technology Research Center for Cardiovascular Disease, Zhujiang Hospital, Southern Medical University, 253# Middle Industrial Avenue, Guangzhou, PR China, 510280.; 2 Key Laboratory of Nephrology, National Health Commission and Guangdong Province; Department of Nephrology, The First Affiliated Hospital, Sun Yat-sen University, 151# Yanjiang Road, Guangzhou, PR China, 510080.; 3 Nanfang Hospital, the First School of Clinical Medicine, Southern Medical University, 1023# Shatai Road South, Guangzhou, PR China, 510515.; 4 State Key Laboratory of Oncology in South China; Collaborative Innovation Center for Cancer Medicine; Guangdong Key Laboratory of Nasopharyngeal Carinoma Diagnosis and Therapy, Sun Yat-sen University Cancer Center, 651# Dongfeng Road East, Guangzhou, PR China, 510060.

**Keywords:** LBH, Exosomes, EMT progression, Angiogenesis, VEGFA, Nasopharyngeal carcinoma

## Abstract

The limb-bud and heart (LBH) gene was reported to suppress nasopharyngeal carcinoma (NPC) progression in our previous study. Distant metastasis predominantly accounts for the unsatisfactory prognosis of NPC treatment, in which epithelial-mesenchymal transition (EMT) and tumor angiogenesis are of great significance. The roles of exosomes in mediating NPC progression have been highlighted in recent researches, and attempts have been made to explore the clinical application of NPC exosomes. Here we investigated the function of the LBH gene in NPC exosomes, and its potential mechanism. NPC xenografts were constructed, showing that vascular endothelial growth factor A (VEGFA) expression and neovascularity were attenuated by LBH overexpression, together with diminished EMT progression. NPC-derived exosomes were isolated, identified and applied for *in vitro/in vivo* experiments, and the exosomal distribution of LBH was elevated in exosomes derived from LBH-upregulated cells. Ectopic LBH, αB-crystallin (CRYAB) and VEGFA expression was induced by lentiviral infection or plasmid transfection to explore their functions in modulating EMT and angiogenesis in NPC. The addition of LBH+ NPC exosomes during a Matrigel plug assay in mice suppressed *in vivo* angiogenesis, and the treatment of human umbilical vein endothelial cells (HUVECs) with LBH+ NPC exosomes inhibited cellular proliferation, migration and tube formation. The interactions among LBH, CRYAB and VEGFA were confirmed by colocalization and fluorescence resonance energy transfer (FRET) assays, and extracellular VEGFA secretion from both HUVECs and NPC cells under the treatment with LBH+ NPC exosomes was diminished according to ELISA results. We concluded that exosomal LBH inhibits EMT progression and angiogenesis in the NPC microenvironment, and that its effects are partially implemented by modulation of VEGFA expression, secretion and related signaling. Thus, LBH could serve as a promising therapeutic target in VEGFA-focused NPC treatment.

## Introduction

Nasopharyngeal carcinoma (NPC) is a squamous cell carcinoma originating from the nasopharyngeal epithelium. Its incidences are mainly geographically distributed in Southeast Asia and Southern China, and remain a serious health concern in endemic regions 1. Although radiotherapy and chemotherapy have improved the general prognosis of NPC patients 2, current screening and diagnostic tools have difficulties detecting NPC at the early stage, and lymph node/distant metastases at the advanced stage usually lead to unsatisfactory treatment outcomes 3. Therefore, the obscure mechanisms underlying NPC progression and metastasis need to be further explored.

Tumor angiogenesis relies on sophisticated cellular activities, including proliferation, migration and differentiation of vascular endothelial cells to form tube structures 4. Tumor microvasculature is essential for the nutrient and oxygen supply to tumor cells; thus, it is crucial for tumor growth and metastasis. For NPC, studies have shown that microvessel density (MVD) is closely correlated with invasion and metastasis detected in tissues from NPC patients 5, 6.

Limb-bud and heart (LBH) is a highly conserved transcription cofactor discovered in embryonic development 7 and has been reported to modulate the progression of various cancer types 8, 9. In a previous study, we demonstrated that LBH participates in regulating tumorigenesis, epithelial-mesenchymal transition (EMT) and metastasis of NPC as a tumor suppressor 10, which encouraged us to further investigate its role during tumor angiogenesis in NPC.

Exosomes are nanoscale multivesicular bodies (MVBs) secreted by almost all cell types, serving as messengers for intercellular communication by delivering functional elements from donor cells to recipient cells, including microRNA/DNA and proteins 11, which affect multiple physiological and pathological processes after internalization by membrane fusion. Specifically, NPC-related exosomes have been reported to mediate cellular proliferation, EMT progression, angiogenesis and metastasis in NPC, and related studies have been applied to clinical diagnosis and therapy 12. The relationship between the LBH gene and NPC-related exosomes, however, has not been studied and requires our examination.

Vascular endothelial growth factor A (VEGFA) is generally considered the primary mediator of angiogenesis 13. VEGFA secretion is highly associated with angiogenesis, metastasis formation and poor prognosis in various cancer types 14, 15. Jiang et al. 16 revealed that LBH modulates angiogenesis in human glioma via VEGFA-mediated signaling. VEGFA was also reported to interact with phosphorylated αB-crystallin (CRYAB) to regulate angiogenesis 17, and our previous study verified that LBH inhibits EMT progression and metastasis in NPC by suppressing CRYAB phosphorylation 10. In our preliminary experiment with NPC cell derived xenografts, LBH overexpression in NPC cells led to inhibited neovascularity and downregulation of CRYAB and VEGFA in harvested xenografts, and LBH protein colocalized with vesicle biomarkers in NPC cells, suggesting potential LBH secretion via exosomes. Based on these reported findings and the results of our preliminary experiment, we aimed to explore the effects and mechanisms of exosomal LBH on EMT progression, metastasis and angiogenesis during NPC development, and the potential relationship between LBH and VEGFA.

## Materials and Methods

### Cell culture and lentivirus infections

Stable cell lines that ectopically express the LBH gene, and their corresponding negative controls were established through lentiviral infection. The NPC cell lines SUNE1 (established at the Sun Yat-sen University), CNE2 (established at the Chinese University of Hong Kong) and Human umbilical vein endothelial cells (HUVECs) were individually infected with Lv5-NC or Lv5-LBH lentivirus (all vectors integrated with eGFP reporter, Genepharma Inc.) in the presence of 0.1% v/v polybrene. After 72 hours, puromycin (2 μg/ml) screening was applied to obtain the stable cell lines SUNE1-Lv5NC, SUNE1-LBH, CNE2-Lv5NC, CNE2-LBH, HUVECs-Lv5NC and HUVECs-LBH. The NPC cell lines were maintained in RPMI 1640 containing 10% fetal bovine serum (FBS) and antibiotics; HUVECs were identified by immunostaining of multiple biomarkers **(Figure S1)** and were maintained in DMEM containing 10% FBS and antibiotics and incubated in standard conditions (37 °C and 5% CO_2_). The lentivirus infection efficiencies were ensured by fluorescence microscopy **(Figure S2)**.

### Exosome isolation and electron microscopy

Exosomes were isolated from NPC cells culture medium by a modified ultracentrifugation method **(Figure S3)**. Briefly, When the cultured NPC cells reached 80% confluency, the culture medium was collected and applied to centrifugation at 500×g for 10 min, 2000×g for 10 min and 10,000 ×g for 30 min at 4 °C (Thermo ST 16R Centrifuge), then the supernatants were applied to centrifugation at 100,000 ×g for 70 min at 4 °C twice (Beckman SW 32Ti Ultracentrifuge), and the pellets were resuspended in 100 μl PBS. For each isolated sample, 50 ml of medium collected from two T75 culture flask was applied to ultracentrifugation. Isolated exosomes were stored at -80 °C and used within a week after isolation. As for electron microscopy, 20 μl/one drop of resuspended exosomes solution was loaded onto 400-mesh carbon-coated copper grids pretreated by glow discharge, and stained by 4% uranyl acetate solution for 10 mins. After washing and air drying, these samples were observed and photographed by a JEM-1400 PLUS (JEOL Co., Ltd, Japan) transmission electron microscope (TEM) equipped with a VELETA G3 CCD.

### Nanoparticle tracking analysis (NTA) and nanoflow cytometry measurement (NFCM)

The Zetaview system (Particle Metrix, Germany) was used for real-time characterization of the NPC-derived exosomes. Samples were diluted 1,000 times for injection, and for each sample, a 20-s video was recorded and analyzed by Software ZetaView version 8.04.02, which were represented as the size distribution of analyzed samples. The flow nano-analyzer (NanoFCM, China) was used for quantification of target protein in NPC-derived exosomes. Isolated samples were stained with fluorescein labelled antibody RT for 30 min after being treated with permeabilization buffer (BD 554714, USA); then, the stained samples were recentrifuged twice at 100,000 ×g for 20 min (Beckman SW 32Ti Ultracentrifuge) to remove any excess unbound antibody and applied for the flow nano-analyzer. The deionized water was used for blank calibration and unstained testing samples were used for negative control.

### Exosome labeling and internalization assay

The exosomes isolated from NPCs were labeled by PKH26 membrane dye (Sigma-Aldrich MINI26, USA) before being applied to the culture medium for NPC cells or HUVECs in order to observe the cellular uptake of labeled exosomes. The labeling methods were performed according to the instructions of the manufacturers. After 3 hours coculture with labeled exosomes (20 μg total protein for each 35 mm petri dish according to BCA assay), treated cells were fixed with 4% paraformaldehyde (PFA) and labeled with phalloidin-FTIC (Sigma-Aldrich P5282, USA). Then, 4',6-diamidino-2-phenylindole (DAPI, FluoroPure™ grade, Invitrogen) was applied to stain the nuclei and the samples were imaged by a Leica SP8 confocal microscope to confirm the internalization and intracellular distribution of labeled exosomes. The 3D images were remodeled from z-stack series with the 3D viewer module of Las X software.

### Cellular proliferation assays

Proliferation experiments were performed by CCK-8 assay, 5-ethynyl-2'-deoxyuridine (EdU) staining and Immunofluorescence staining of anti-Ki67. The CCK-8 assay was performed by CCK-8 assay kit (Dojindo Inc.) and measured by a Varioskan LUX plate reader (Thermo Scientific) based on a previously reported protocol 18. The EdU staining was performed with BeyoClick^TM^ EdU-555 assay kit (Beyotime Biotech), all according to the instructions of the manufacturers. Briefly, complete culture medium was replaced by staining medium containing 10 μM EdU; 3 hours later, the cell samples were fixed by 4% PFA for 10 min, treated by 0.3% Triton X-100 for 10 min, and incubated by labeling buffer RT for 30 min. Then, the samples were stained by DAPI and imaged by a Leica DMi8 fluorescence microscope. The EdU positive rates and Ki67 positive rates were calculated by ImageJ software, and for every sample the EdU/Ki67 positive rates of 6 random fields were used for statistics.

### Cellular migration/ Invasion assay

Cells were serum starved overnight (O/N) before the experiment. Then the serum-free cell suspensions were seeded into the transwell inserts (Corning #3422, 8.0 μm pore size) at a density of 150,000 cells/100 μl/insert for migration assays, or into Matrigel-coated inserts (Corning #354480, 8.0 μm pore size) for invasion assays, and culturing medium containing 20% FBS was added into each lower chamber. For NPC cells, the inserts were fixed with 4% PFA, and stained with 2.5% crystal violet for 10 min 24 hours later, and for HUVECs the inserts were sampled 12 hours after seeding. Cells attached to the lower sides of insert membranes were visualized as migrated or invaded cells and photographed by a Leica DMi8 microscope. For each cell line, the numbers of migrated/invaded cells in six individual fields were counted by ImageJ software and used for statistical analysis.

### Tube formation assay

50 μl precooled Matrigel (Corning #356234) were coated into each well of a precooled 96-well plate and incubated at 37 °C, while HUVECs were serum starved. 6 hours later, HUVECs were detached and resuspended in DMEM containing 2% FBS. Every 20,000 HUVECs in 150 μl cell suspension containing 1 μg isolated exosomes were seeded into each well. After 9 hours, tube-like structures were photographed by a Leica DMi8 microscope and the images were analyzed by an Angiogenesis Analyzer plugin 19 of the ImageJ software.

### Quantitative real-time PCR

Both the treated Cells and the isolated exosomes were sampled with TRIzol^®^ reagent (Invitrogen). The reverse transcription was performed with a PrimeScript^TM^ RT reagent kit (Takara), and the qPCR procedure was performed with a SYBR^®^ Premix Ex Taq ^TM^ kit (Takara), all according to the instructions of the manufacturers. The primer sequences (synthesized by Takara Bio Inc.) are shown in **Table S1.** qPCR assays were conducted on an Applied Biosystem 7500 Fast instrument. The mRNA transcription levels of target genes were quantified by 2^-ΔΔCt^ method, with GAPDH as house-keeping gene for cell samples and CD63 for exosomes samples.

### Animal procedures

All animal procedures were designed and performed according to the regulations of the Institutional Animal Care and Use Ethics Committee of Zhujiang Hospital. The BALB/c nude mice were purchased from the Medical Experimental Animal Center of Guangdong Province and maintained at the Animal Experiment Center of Zhujiang Hospital. For xenograft tumor models, 16 nude mice (4 weeks, male) were randomly divided into four groups (n=4 per group) and 5,000,000 CNE2-Lv5NC, CNE2-LBH, SUNE1-Lv5NC or SUNE1-LBH cells in 200 μl suspension were injected into the dorsal flank of the mice. 4 weeks later, the mice were sacrificed, and their tumor xenografts were harvested. For Matrigel plus assay, 8 nude mice were randomly divided into two groups (n=4 per group); then, for each mouse 500 μl growth factor reduced Matrigel (Corning #356231) mixed with NPC exosomes (40 μg total protein) and 1,000,000 HUVECs were slowly injected subcutaneously into the dorsal flank and allowed to solidify 20. 2 weeks later, the mice were sacrificed, and their Matrigel plugs were harvested. All these samples were paraffin-embedded, sectioned and applied to the following hematoxylin-eosin (H&E) staining, immunofluorescence staining, or magnetically homogenized by a tissue homogenizer (Jingxin Tissuelsyer-24) and applied for the western blot assays.

### Western blot assays

Western blotting assays in this study were performed as previously described 21. Cells were sampled in RIPA^TM^ (Thermo Scientific) lysis buffer after 24 hours exosomes treatment. Lysates were probe sonicated and centrifuged (12000 rpm at 4 °C for 15 min). A Pierce^®^ BCA assay (Thermo Scientific) was used to determine the protein concentrations of cell lysates and isolated exosomes. 30 μg of each cell lysate sample was loaded into SDS-PAGE gels and electrophoresed, while for exosome samples the loading amount was 60 μg after being concentrated by a freeze dryer (Chirst ALPHA 1-4 LD plus). The separated proteins were blotted onto a 0.22 μm nitrocellulose membrane and blocked with 5% w/v BSA in TBS solution for 2 hours. Primary antibody incubations were then performed with antibody working solutions in 5% w/v BSA in TBST at 4 °C O/N, followed by incubation with secondary antibody in TBST antibody working solutions at RT for 2 hours. The antibodies used are listed in **Table S2**. Stripping buffer (Thermo Scientific) was used to retrieve the membranes between individual primary antibody incubations, membrane exposure was performed by ECL, and a GE ImageQuant LAS 500 exposure instrument, while the quantifications were performed by ImageJ software.

### Immunofluorescence staining

Immunofluorescence staining were performed as previously described 21. Cell samples were then fixed by 4% PFA for 10 min. Tissue sections received deparaffinization (Histoclear, National Diagnostics), rehydration and sodium citrate antigen retrieval. All samples were blocked with 10% goat serum for 30 min, incubated with primary antibodies O/N at 4 °C and then incubated with fluorescence-conjugated secondary antibodies at room temperature for 2 hours. Then, DAPI was applied, the samples were mounted and then imaged by a Leica SP8 confocal fluorescence microscope. The co-localization ratios of dual staining images were calculated by the Analysis module of Leica Application Suite X software. The whole-slide images of mounted tissue sections were photographed by a GE Amersham Typhoon imager. The in cell western (ICW) assay was performed with a modified protocol based on the procedures of Egorina et al. 22 and photographed by GE Amersham Typhoon imager. The microvessel density (MVD) were quantified by 100-fold anti-CD34 staining images of tumor xenografts or Matrigel plugs, as previously described by Foote et al. 23. For each sample, they were divided into three layers and tissue slices from each layer were taken into analysis 24. The vessels in “vascular hot spots” are counted, and presented as numbers per mm^2^ 25.

### Enzyme-linked immuno-sorbent assay (ELISA)

The VEGFA secretion from NPC cells and HUVECs were measured by a human VEGF sandwich ELISA kit (Proteintech, KE00085). Briefly, the treated SUNE1 and HUVEC cells, and their negative controls were seeded in equal numbers; then they were serum starved O/N, washed and incubated with fresh serum free medium for additional 24 hours before the supernatants were sampled by centrifugation and applied to ELISA tests, which were performed all according to the instructions of the manufacturers. For each T25 flask 5ml culture medium were collected; the supernatants were diluted 2 or 4 times to ensure the standard curves were applicable for all samples.

### Plasmid transfection

The dual-plasmid co-transfection prepared for FRET assay were performed with liposomes (Lipofectamine 3000, Invitrogen) according to the manufacturer's instructions. The plasmids expressing fusion proteins were constructed as: pEGFP-C_1_-LBH for expressing LBH-eGFP, pLV-CMV-CRYAB-mCherry-6His-IRES-Bla for expressing CRYAB-mCherry, while pcdh-cmv-VEGFA-eGFP-ef1-puro and pcdh-cmv-VEGFA-mCherry-ef1-puro for expressing VEGFA-eGFP and VEGFA-mCherry. Correspondingly, plasmid pEGFP-C_1_, pLV-CMV-mCherry-6His-IRES-Bla, pcdh-cmv-eGFP-ef1-puro and pcdh-cmv-mCherry-ef1-puro were used as negative controls. The VEGFA plasmids were constructed to express VEGFA_165_, which is considered the predominant isoforms HUVECs during angiogenesis 26, 27, and the sequences were synthesized based on NCBI sequence NM_001025366.3. The ability of these plasmids to overexpress the target genes were also confirmed before performing the FRET assay **(Figure S4).**

### Fluorescence resonance energy transfer

The FRET assay was used to confirm the protein-protein interaction (PPI) among LBH, CRYAB and VEGFA in HUVECs and was designed based on a previously described protocol 28. According to the requirements for sensitized emission (SE) method, for each pair of proteins, 4 groups were established for plasmid transfection: negative control, donor only, acceptor only, which were designed for calibrations, and the dual-transfected group for the FRET assay. The calculations of the FRET efficiencies were performed by the FRET-SE module of Leica Application Suite X software, based on an established formula 29:



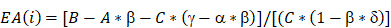



*A,* Donor channel; *B,* FRET channel, *C,* Acceptor channel; *α = A/C*; *β=B/A*; *γ=B/C*; *δ=A/B.*

### Statistical analysis

The data presented were collected from three independent, parallel experiments, and are presented as the mean ± SEM. Statistical analysis was conducted using unpaired Student's t-tests and one-way ANOVA (Tukey's test), with GraphPad Prism software V7.0. p values < 0.05 were considered statistically significant.

## Results

### LBH upregulation is associated with attenuated angiogenesis, EMT progression and VEGFA expression in NPC tumor xenografts

**Figure 1:**
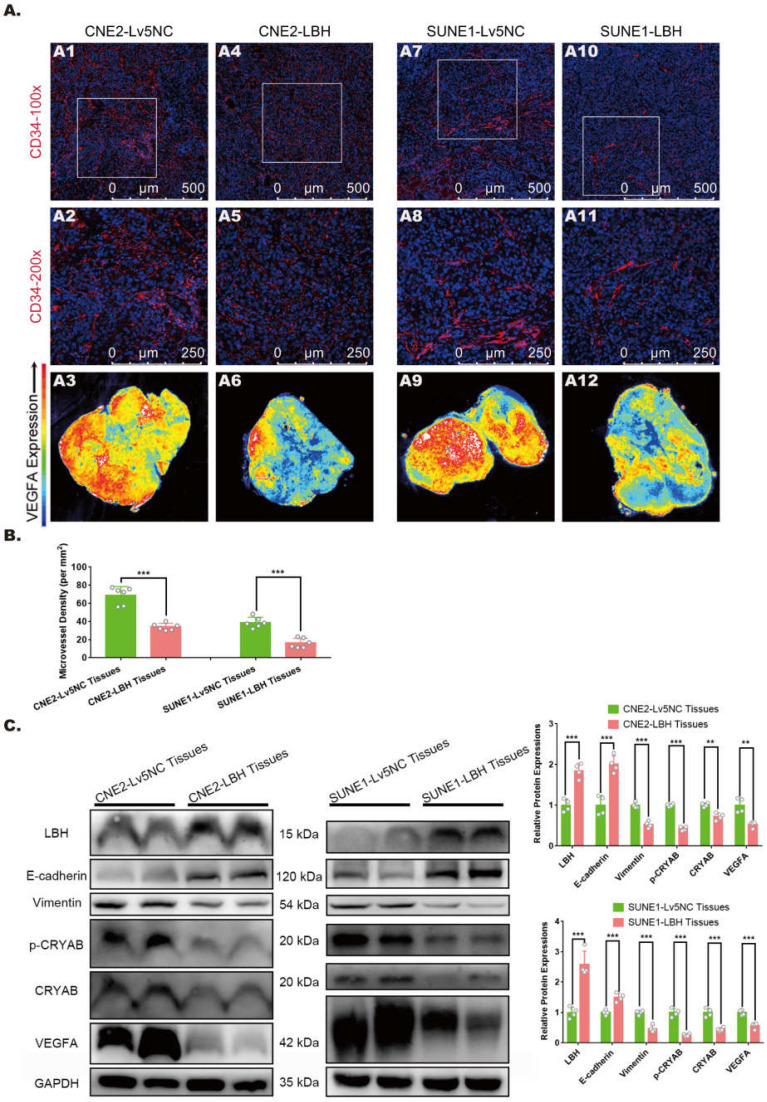
** LBH overexpression is correlated with inhibited EMT progression, angiogenesis, and VEGFA expression in NPC xenograft tumors. (A)** Representative immunofluorescence images of CD34 and VEGFA expression in tumor xenografts constructed with CNE2-Lv5NC, CNE2-LBH, SUNE1- Lv5NC, or SUNE1-LBH cells. The white boxes in 100-fold images of CD34 staining indicate the areas of the 200-fold CD34 staining images for each presented tissue. **(B)** Microvessel densities of tumor xenograft tissue slides, presented as numbers per mm^2^ (***p<0.001 vs. Lv5NC). **(C)** Protein expression of LBH, E-cadherin, Vimentin, p-CRYAB, CRYAB and VEGFA in the same tumor xenografts (*p<0.05, **p<0.01 and ***p<0.001 vs. Lv5NC).

In our previous study, LBH was reported to inhibit EMT progression in nasopharyngeal carcinoma by downregulating CRYAB expression, and its effect on mitigating tumor growth was verified in xenografts constructed with multiple NPC cell lines 10. The growth and metastasis of solid tumors rely on the microvasculature in the tumor microenvironment, and neovascularity, in which VEGFA is considered to play crucial roles, has frequently been discussed as a potential indicator of tumor subtype or stage 30, 31. Thus, we explored whether the LBH gene is correlated with EMT, angiogenesis and VEGFA expression in NPC tumor xenografts. Immunostaining with anti-CD34 showed that in both CNE2 and SUNE1 tumor xenografts, tumors constructed with LBH-overexpressing cell lines presented markedly decreased MVDs in vascular hot spots compared to the negative controls **(Figure 1A, B)**, indicating attenuated angiogenesis in LBH-overexpressing tumors. Then the same tissues were subjected to western blotting, and we found that LBH upregulation significantly decreased the protein expression of Vimentin, p-CRYAB and CRYAB, and increased E-cadherin expression **(Figure 1C)**, showing inhibited EMT progression, which is consistent with our previous results in NPC cells. LBH upregulation also significantly decreased VEGFA expression, which was verified by whole-slide images of anti-VEGFA staining **(Figure 1A, C)**, whose upregulation and secretion were generally considered pro-angiogenic 32, 33. Moreover, H&E staining indicated that for both CNE2 and SUNE1 NPC xenografts, the tissues of LBH-overexpressing tumors were well-differentiated compared to the controls, exhibiting relatively epithelial characteristics, while the tissues of Lv5NC tumors showed poorly-differentiated, relatively mesenchymal characteristics **(Figure S5)**. Since cancer studies almost exclusively characterize EMT as dedifferentiation 34, the differentiation promoted by the LBH gene could be considered to indicate EMT inhibition in LBH-overexpressing NPC xenografts. Thus, we reached the conclusion that the overexpression of the LBH gene is associated with attenuated angiogenesis, EMT progression and VEGFA expression in NPC tumor xenografts.

### Exosomal distribution of LBH protein is elevated in exosomes secreted by LBH-overexpressing NPC cells

**Figure 2:**
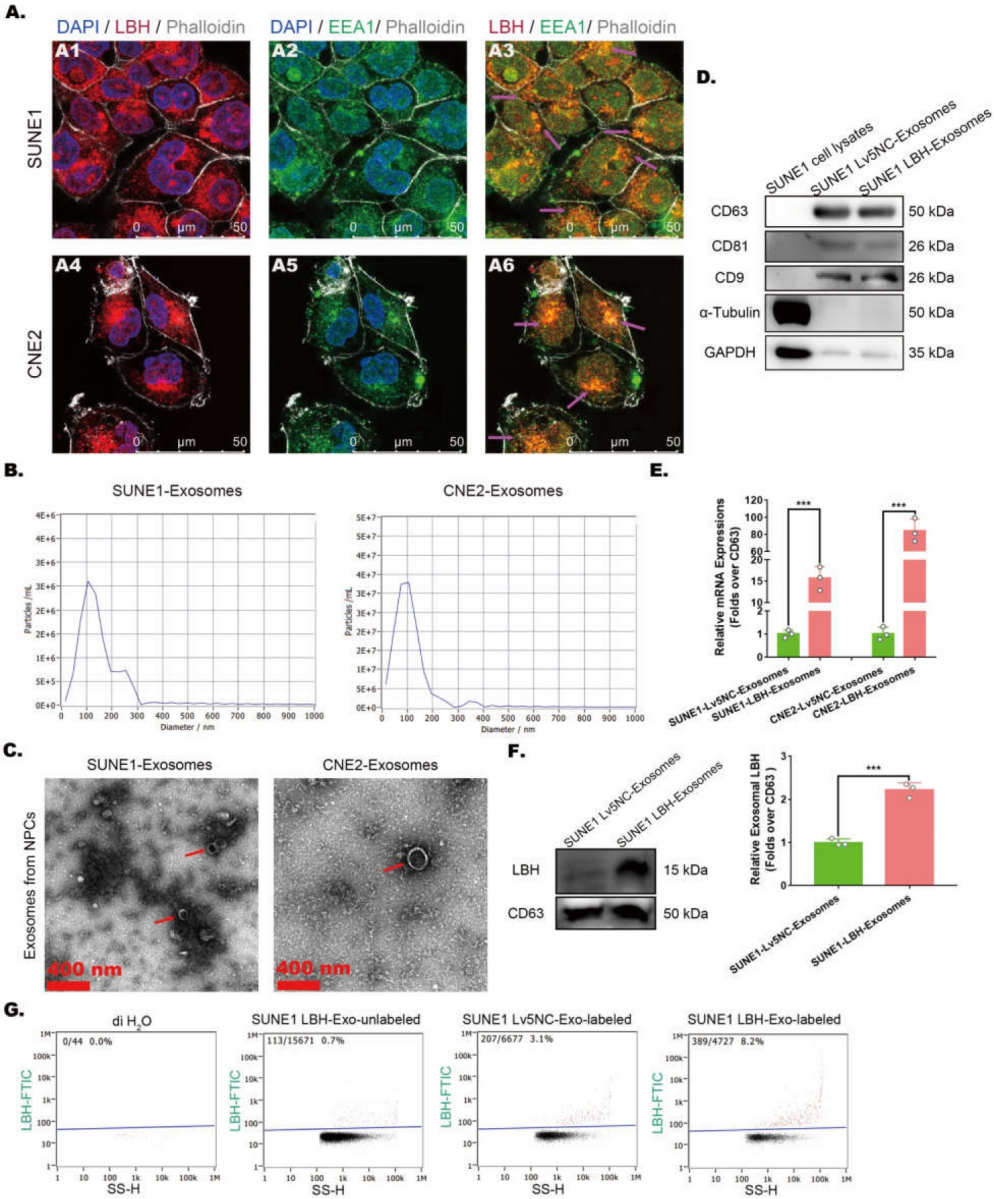
** Exosomal distribution of LBH protein is elevated in exosomes secreted by LBH-overexpressing NPC cells. (A)** Representative confocal microscopy images of dual staining with anti-LBH (red) and anti-EEA1 (green) in CNE2 and SUNE1 cells. Colocalization of LBH and EEA1 was observed in the perinuclear cytoplasm of both CNE2 and SUNE1 cells (indicated by magenta arrows).** (B)** The distribution of particle size/concentration of exosome samples analyzed by NTA assay. **(C)** Representative TEM images of exosome samples derived from CNE2 and SUNE1 cell lines (exosomal structures were indicated by red arrows). **(D)** Protein expression of exosome markers (CD9, CD63, CD81) in both NPC cell lysates and exosomes secreted by NPC cells. **(E)** mRNA levels of LBH in exosomes secreted by NPC cells. **(F)** Western blotting measuring LBH protein in SUNE1^LBH+^ exosomes and SUNE1^NC^ exosomes. **(G)** SUNE1-Lv5NC exosome samples and SUNE1-LBH exosome samples were tested by NanoFCM flow nano-analyzer after being stained with anti-LBH-FITC.

The effects of LBH overexpression on attenuated angiogenesis in NPC xenografts were achieved by crosstalk between NPC cells and HUVECs, in which exosomes might be involved by transporting functional elements. In NPC cells, LBH was observed to colocalize with early endosome antigen 1 (EEA1) **(Figure 2A)**, which implies possible LBH secretion via exosomes. Thus, exosomes derived from NPC cell lines were isolated by differential ultracentrifugation, and a combination of tests were performed to identify the isolated samples as exosomes. First, NTA analysis revealed the major components of isolated samples with average sizes of 120.1 nm (sample from CNE2 cells) and 95.5 nm (sample from SUNE1 cells) **(Figure 2B)**; then, clear lipid bilayer membranes with major diameters of 100-200 nm were observed in TEM images **(Figure 2C)**. Finally, the isolated samples showed higher protein levels of exosome markers CD9, CD63 and CD81, as well as lower levels of housekeeping genes in western blotting compared to NPC cell lysates **(Figure 2D)**. In addition, SUNE1-LBH exosomes exhibited a higher LBH distribution than SUNE1-Lv5NC exosomes at both the mRNA **(Figure 2E)** and protein levels **(Figure 2F, G)**. Collectively, these data suggest that we successfully isolated exosomes from the culture medium of NPC cell lines and that the exosomal distribution of LBH protein is elevated in exosomes secreted by SUNE1-LBH cells compared to SUNE1-Lv5NC cells.

### LBH upregulation via exosome internalization modulates EMT progression in NPC cells by downregulating VEGFA

**Figure 3:**
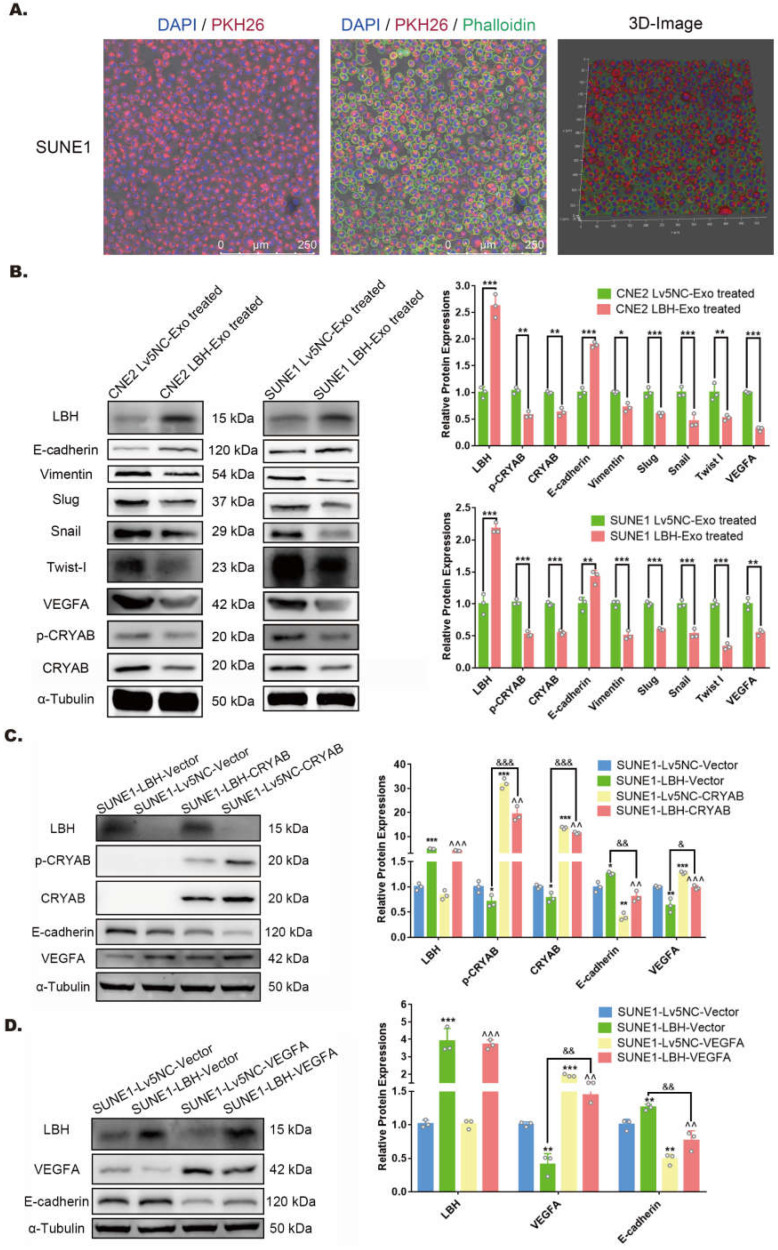
** LBH upregulation in NPC cells is implemented by the internalization of NPC^ LBH+^ exosomes, and inhibits EMT progression via downregulating VEGFA in NPC cells. (A)** Representative confocal microscopic images of SUNE1 cells treated with PKH26-labeled NPC exosomes. **(B)** Protein levels of LBH, Vimentin, E-cadherin, p-CRYAB, CRYAB, VEGFA, Snail, Slug and Twist I in NPC cells treated with NPC exosomes (*p<0.05, **p<0.01 and ***p<0.001 vs. Lv5NC-Exo treated). **(C)** Protein levels of LBH, p-CRYAB, CRYAB, VEGFA and E-cadherin in LBH overexpressing SUNE1 cells followed by CRYAB plasmid transfection (*p<0.05 and **p<0.01 vs. SUNE1-Lv5NC-Vector; ^^p<0.01 and ^^^p<0.001 vs. SUNE1-Lv5NC-CRYAB; ^&^p<0.05 and ^&&&^p<0.001 vs. SUNE1-LBH-Vector). **(D)** Protein levels of LBH, VEFGA and E-cadherin in LBH-overexpressing SUNE1 cells followed by VEGFA plasmid transfection (**p<0.01 and ***p<0.001 vs. SUNE1-Lv5NC-Vector; ^^p<0.01 and ^^^p<0.001 vs. SUNE1-Lv5NC-VEGFA; ^&&&^p<0.001 vs. SUNE1-LBH-Vector).

NPC-derived exosomes have been reported to orchestrate both autocrine and paracrine functions in the microenvironment to regulate tumor progression 35. SUNE1 cells were cocultured with PKH26-labeled exosomes previously isolated from the same cells, and showed considerable cellular uptake of the labeled exosomes **(Figure 3A)**. Thus, to determine the potential effects of LBH+ exosomes on NPC cells after internalization, both CNE2/SUNE1^NC^ exosomes and CNE2/SUNE1^LBH+^ exosomes were cocultured with CNE2 or SUNE1 cells respectively. The results showed that in both CNE2 and SUNE1 cells, coculture with LBH+ exosomes upregulated the expression of LBH and E-cadherin, while downregulating CRYAB and Vimentin expression at the mRNA and protein levels compared to cells cocultured with NC exosomes **(Figure 3B, Figure S6B)**; synchronously, diminished expression of p-CRYAB, VEGFA, EMT-TFs Snail, Slug and Twist I, together with enhanced E-cadherin expression was revealed by western blotting **(Figure 3B)**. This was in accordance with our previous results in stable LBH-overexpressing NPC cell lines 10. In addition, CRYAB upregulation by plasmid transfection was accompanied by enhanced VEGFA expression and EMT progression, which were partially reversed by LBH overexpression **(Figure 3C)**; VEGFA upregulation by plasmid transfection was correlated with promoted EMT progression, and LBH overexpression also partially reversed this trend **(Figure 3D)**. These results validated that in the NPC microenvironment, LBH upregulation in NPC cells leads to augmented exosomal distribution of LBH protein, and that cellular internalization of LBH+ exosomes increases LBH levels in NPC cells, which downregulates VEGFA expression by modulating CRYAB phosphorylation. This VEGFA downregulation is correlated with inhibited EMT progression in NPC cells induced by LBH+ exosomes.

### LBH upregulation diminishes the migration and invasion of NPC cells and angiogenic capacity of HUVECs via exosomes

**Figure 4:**
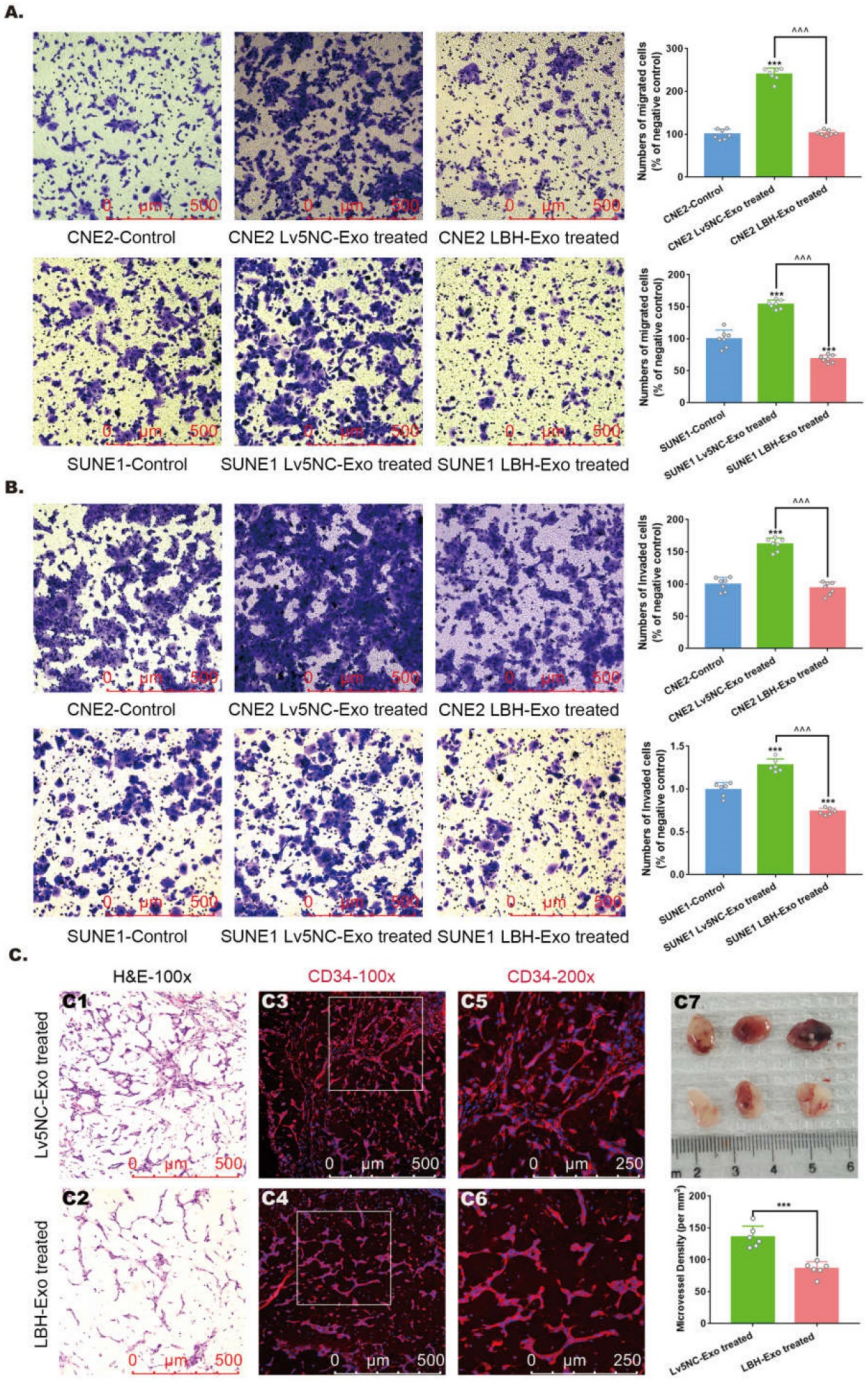
** The effects of the internalization of NPC^ LBH+^ exosomes on cellular migration, invasion and angiogenesis of nasopharyngeal carcinoma.** Representative images of the Transwell assay (**A**) and Matrigel Transwell assay (**B**) of NPC cells treated with NPC exosomes, and corresponding statistical analysis (***p<0.001 vs. Control; ^^^p<0.001 vs. Lv5NC-Exo treated). **(C)** Representative images of H&E staining and CD34 immunofluorescence staining in exosome-treated Matrigel plugs planted in nude mice, and microvessel densities of corresponding tissue slides, presented as numbers per mm^2^ (***p<0.001 vs. Lv5NC-Exo treated).

To further investigate the roles of NPC^LBH+^ exosomes in NPC metastasis and angiogenesis, assays testing the migration and invasion of NPC cells, as well as neovascularity were conducted *in vitro* and *in vivo*. LBH overexpression has been confirmed to diminish the migrating and invading capacities of CNE2 cells in our previous study 10, and here we proved that it was also applied to SUNE1 cells **(Figure S7)**. In addition, Transwell assays and Matrigel Transwell assays showed reduced migrated and invaded cell counts for LBH+ exosome-treated CNE2/SUNE1 cells compared with NC exosome-treated CNE2/SUNE1 cells **(Figure 4A, B)**. For angiogenesis, we found that treatment with SUNE1^LBH+^ exosomes significantly attenuated vessel formation in a Matrigel plug assay compared to treatment with SUNE1^NC^ exosomes, which was indicated by H&E staining and decreased MVDs calculated based on anti-CD34 staining of Matrigel plugs synchronously implanted with NPC exosomes and HUVECs **(Figure 4C)**. Conclusively, LBH overexpression in NPC cells caused elevated LBH levels in NPC-derived exosomes, and upon internalization, these LBH+ exosomes diminished the migration and invasion of NPC cells and angiogenic capacity of HUVECs.

### Exosomes secreted by LBH-overexpressing NPC cells inhibit the proliferation, migration and tube formation of HUVECs *in vitro*

**Figure 5:**
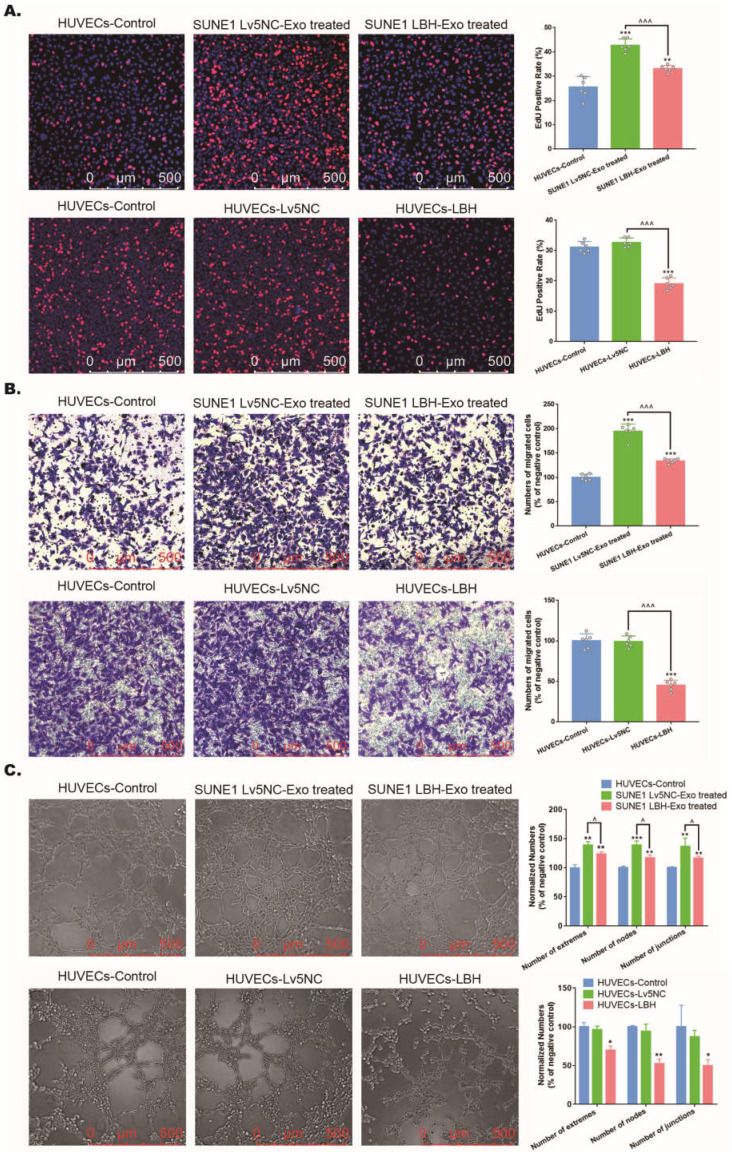
** The effects of the internalization of SUNE1^LBH+^ exosomes and LBH overexpression on the cellular proliferation, migration and tube formation of HUVECs. (A)** EdU staining of HUVECs treated with NPC exosomes and stable LBH overexpressing HUVECs (**p<0.01 and ***p<0.001 vs. Control; ^^^p<0.001 vs. Lv5NC/Lv5NC-Exo treated). **(B)** Transwell assay of HUVECs treated by NPC exosomes and stable LBH overexpressing HUVECs (***p<0.001 vs. Control; ^^^p<0.001 vs. Lv5NC/Lv5NC-Exo treated). **(C)** Tube formation assays of HUVECs treated with NPC exosomes and stable LBH overexpressing HUVECs (*p<0.05 and **p<0.01 vs. Control; ^p<0.05 vs. Lv5NC/Lv5NC-Exo treated).

Based on the effects of SUNE1^LBH+^ exosomes on vessel formation, we went one step further to investigate its potential involvement of angiogenesis in NPC tumors, which is predominantly implemented by the proliferation, migration and tube formation of HUVECs 36. Firstly, we confirmed that exosomes derived from SUNE1 cells could be internalized by HUVECs **(Figure S8)**. Then, we found that treatment with SUNE1^LBH+^ exosomes diminished HUVEC proliferation compared to treatment with SUNE1^NC^ exosomes, which was similar to the effects of LBH overexpression in HUVECs; while coculture with SUNE1^NC^ exosomes increased HUVEC proliferation compared to the negative controls **(Figure 5A)**. Since EdU staining indicates only the S phase in the cell cycle as a proliferation marker 37, CCK-8 assays and anti-Ki67 staining were also applied, and both of them presented the same results **(Figure S9)**. Correspondingly, treatment with SUNE1^LBH+^ exosomes inhibited the migration and tube formation of HUVECs compared to treatment with SUNE1^NC^ exosomes, and LBH overexpression led to similar results; while coculture with SUNE1^NC^ exosomes promoted HUVEC migration and tube formation compared to the negative controls **(Figure 5B, C)**. These data uniformly confirmed that exosomes secreted by LBH-overexpressing NPC cells inhibit the proliferation, migration and tube formation of HUVECs *in vitro*, while their effects on NPC tumors *in vivo* need further investigation.

### Exosomes secreted by LBH-overexpressing NPC cells regulate VEGFA/VEGFR2 signaling in HUVECs

**Figure 6:**
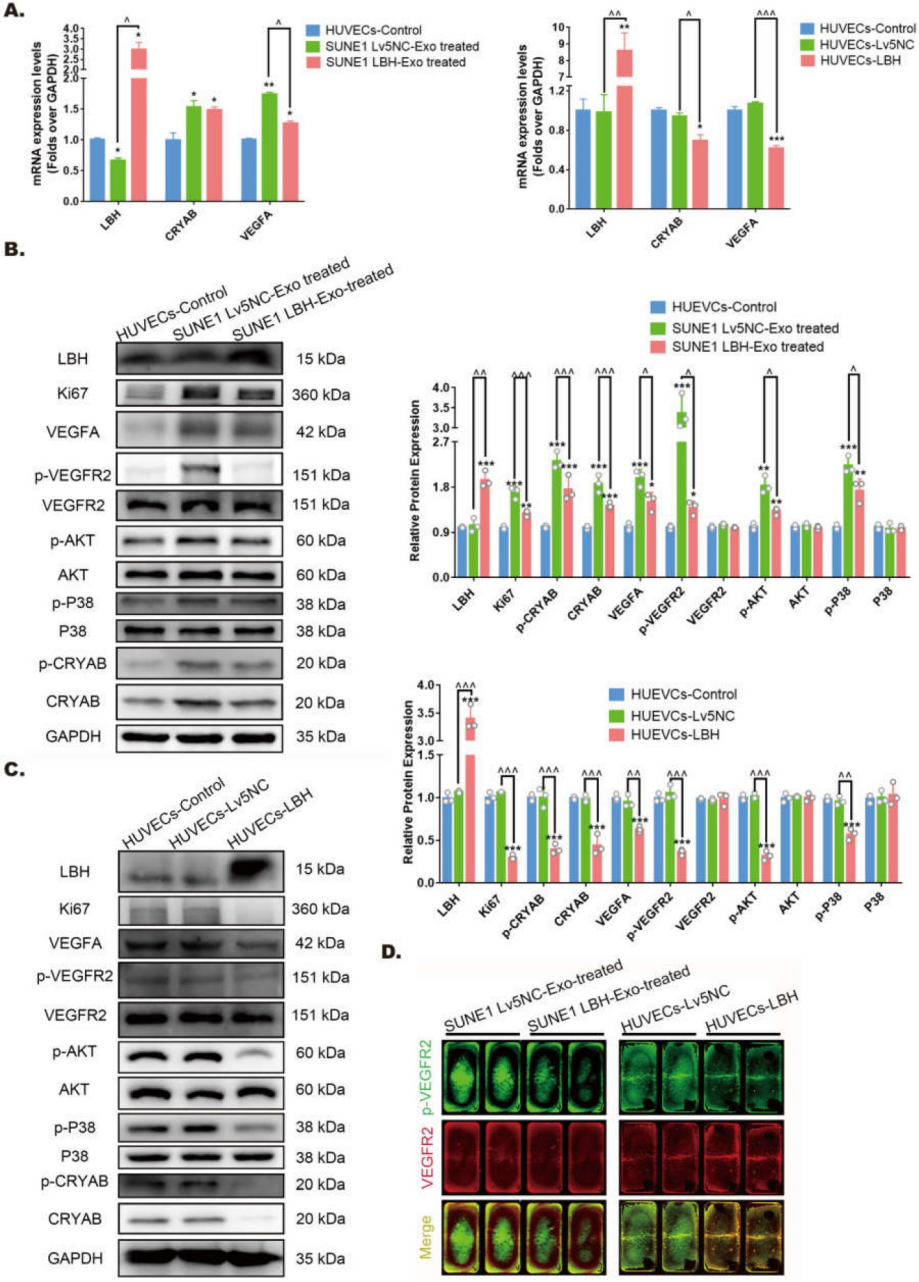
** Both the internalization of SUNE1^LBH+^ exosomes and the LBH overexpression regulate VEGFA/VEGFR2 signaling in HUVECs. (A)** mRNA levels of LBH, CRYAB and VEGFA in HUVECs treated with NPC exosomes and stable LBH overexpressing HUVECs (*p<0.05 and **p<0.01 vs. Control; ^p<0.05 vs. Lv5NC/Lv5NC-Exo treated). Protein levels of LBH, Ki67, p-CRYAB, CRYAB, VEGFA, p-VEGFR2, VEGFR2, p-AKT, AKT, p-P38, and P38 in HUVECs treated with NPC exosomes (**B**) and stable LBH overexpressing HUVECs (**C**) (*p<0.05, **p<0.01 and ***p<0.001 vs. Control; ^p<0.05, ^^p<0.01 and ^^^p<0.001 vs. Lv5NC / Lv5NC-Exo treated). **(D)** In cell western simultaneously testing the protein levels of p-VEGFR2 (green) and VEGFR2 (red) in HUVECs treated with NPC exosomes and stable LBH overexpressing HUVECs.

Our previous research indicated that ectopic LBH expression inhibits the phosphorylation of CRYAB 10. Also, CRYAB phosphorylation has been reported to be associated with its ability to interact with misfolded VEGFA protein and thus leads to increased VEGFA secretion and promoted angiogenesis 38, manifesting as enhanced proliferation, migration and tube formation of HUVECs. Since we found that treatment of HUVECs with SUNE1^LBH+^ exosomes caused LBH upregulation and VEGFA downregulation at mRNA levels compared to HUVECs cocultured with SUNE1^NC^ exosomes **(Figure 6A)**, the potential activation of downstream effectors of VEGFA/VEGFR2 signaling in NPC exosome-treated HUVECs were further examined. The results showed that coculture with SUNE1^LBH^ exosomes decreased the expression of p-CRYAB, CRYAB and VEGFA, together with inhibited phosphorylation of VEGFR2, P38 and AKT in HUVECs compared to treatment with SUNE1^NC^ exosomes **(Figure 6B, D)**, which was similar to the effects of LBH overexpression in HUVECs **(Figure 6A, C, D)**. In addition, compared to the negative controls, coculture with SUNE1^NC^ exosomes significantly upregulated CRYAB and VEGFA in HUVECs at both the mRNA and protein levels, which was accompanied by augmented phosphorylation of CRYAB, VEGFR2, P38 and AKT **(Figure 6A, B, D)**. The phosphorylation levels of ERK1/2 and Smad3 in HUVECs, however, remained stable during both exosome treatments and lentiviral overexpression of LBH **(Figure S10)**. Furthermore, we also upregulated CRYAB in HUVECs by plasmid transfection, and ectopic CRYAB expression led to increased p-CRYAB and VEGFA levels, which verified that VEGFA is regulated by CRYAB in HUVECs **(Figure S11)**. Altogether, these results suggest that exosomes secreted by LBH-overexpressing NPC cells induce LBH upregulation in HUVECs and subsequently suppress VEGFA/VEGFR2 signaling by downregulating CRYAB expression and phosphorylation.

### Protein-protein interactions among LBH, CRYAB and VEGFA in HUVECs

**Figure 7:**
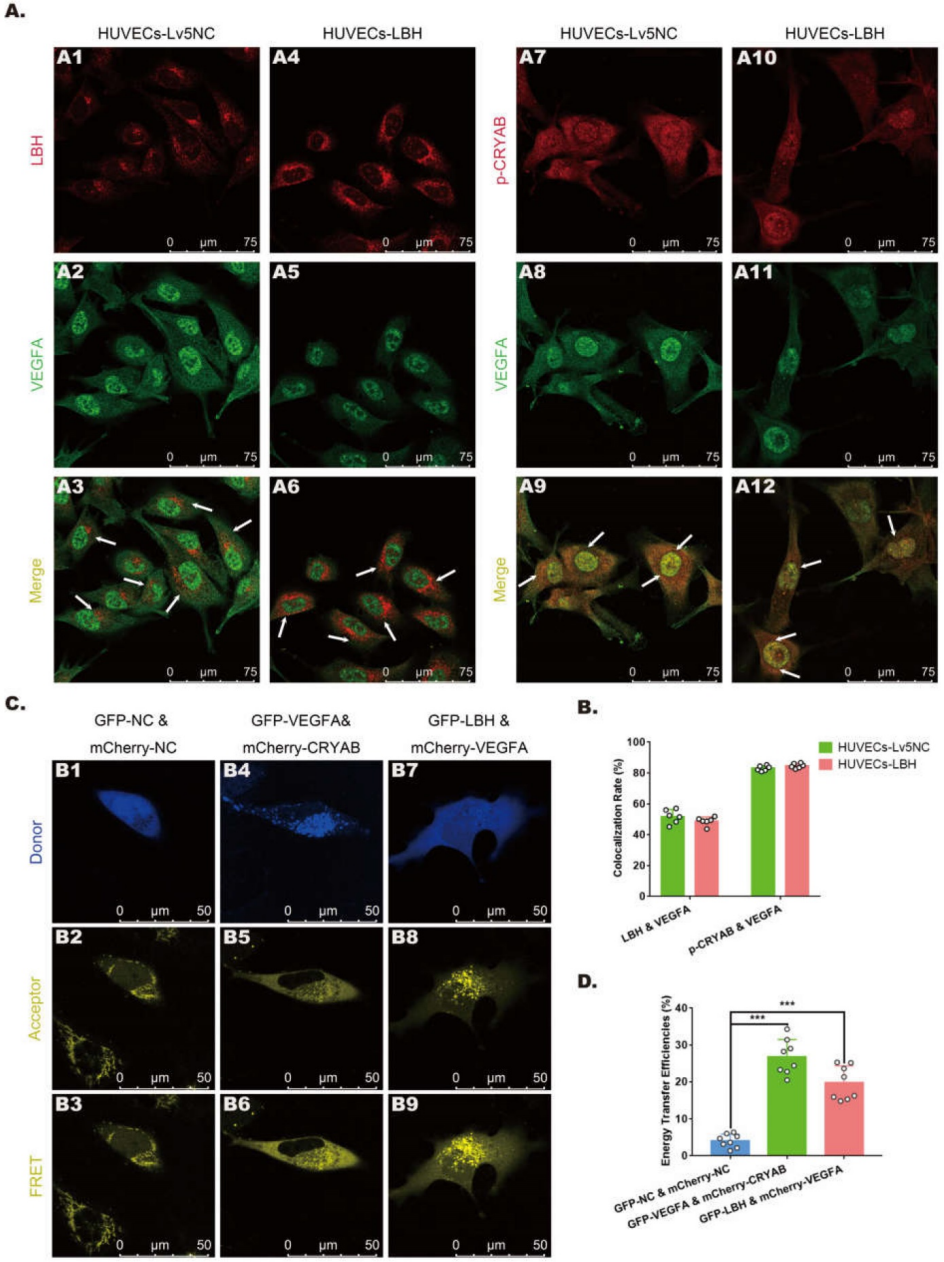
** The protein-protein interactions among LBH, CRYAB and VEGFA in HUVECs. (A)** Representative confocal microscopy images of LBH (red) and VEGFA (green), or p-CRYAB (red) and VEGFA (green) dual staining in stable LBH overexpressing HUVECs, and quantification of the colocalization ratios **(B)** of these double-stained HUVECs. The colocalizations in subcellular regions are indicated by white arrows. **(C)** Representative images obtained by the FRET-SE pattern on a Leica SP8 confocal microscope, and the corresponding statistical analysis of the FRET efficiencies **(D)** for the negative control, for GFP-LBH & mCherry-VEGFA and for GFP-VEGFA & mCherry-CRYAB in HUVECs (***p<0.001 vs. GFP-NC & m Cherry-NC).

In our previous study, CRYAB was identified as an LBH-interacting protein in NPC cells using a FRET assay 10, while it has also been reported as a VEGFA chaperone regulating angiogenesis in retinal pigment epithelial cells 38. To explore the relationships among LBH, CRYAB and VEGFA in HUVECs, fluorescence colocalization and FRET assays were designed to evaluate possible protein-protein interactions. Confocal microscopy images **(Figure 7A)** showed colocalization of LBH and VEGFA, and of CRYAB and VEGFA in both the nucleus and cytoplasm in HUVECs, and the calculated colocalization ratios were considerable** (Figure 7B).** The plasmids verified by western blotting **(Figure S4)** were transfected into HUVECs for FRET assay, and the results indicated that co-transfection of HUVECs with LBH-GFP and VEGFA-mCherry induced significantly higher average FRET efficiencies than those of the negative controls, and the same results were obtained from co-transfection of HUVECs with VEGFA-GFP and CRYAB-mCherry (**Figure 7C, D**). The validated FRET effects between LBH and VEGFA, or between CRYAB and VEGFA could be viewed as direct evidence of PPIs among these proteins in HUVECs.

### The effects of elevated LBH expression on VEGFA secretion and VEGFA/VEGFR2 signaling in NPC

**Figure 8:**
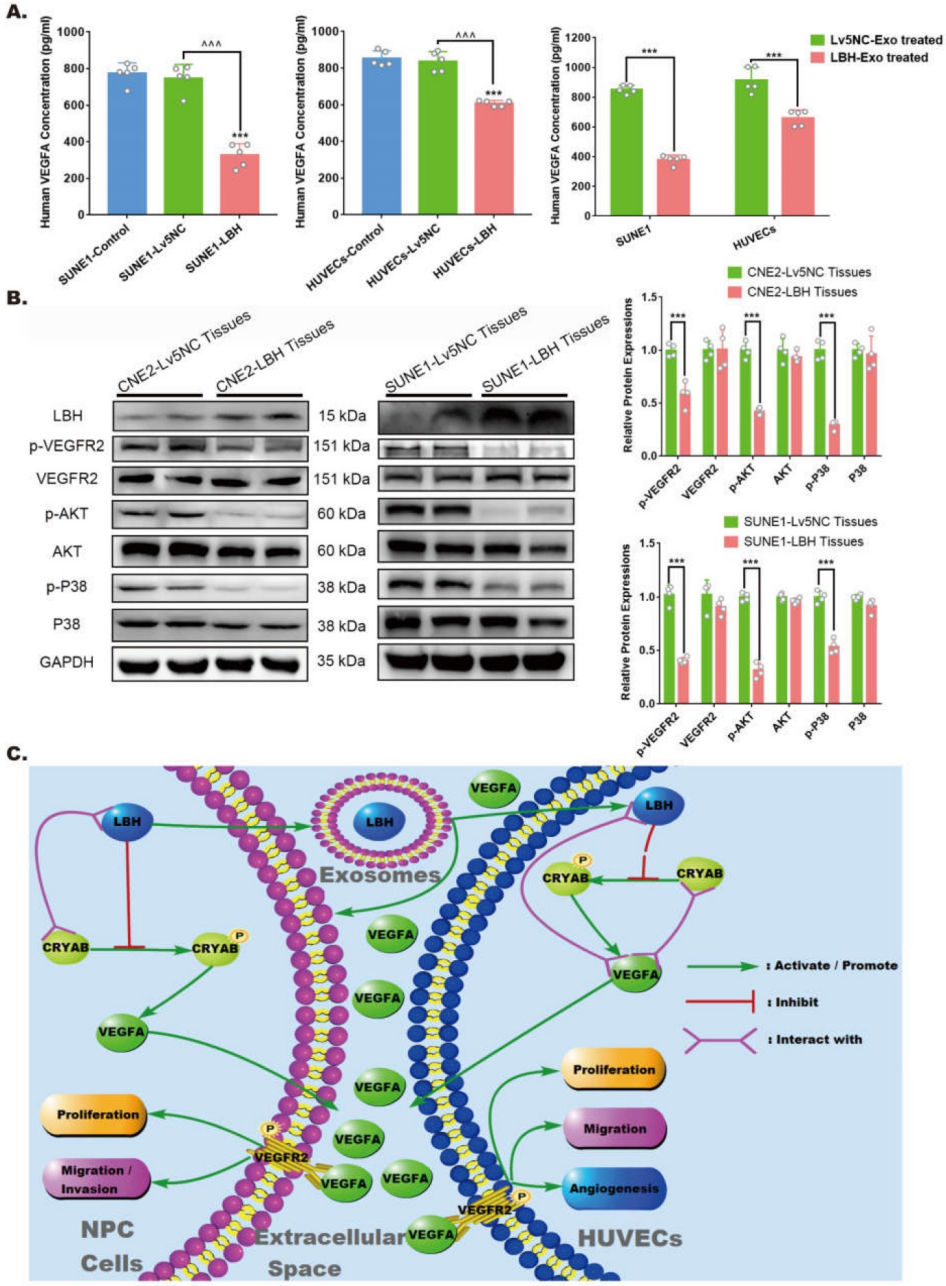
** LBH overexpression suppressed VEGFA secretion from both NPC cells and HUVECs, and downregulated VEGFA/VEGFR2 signaling in NPC xenograft tumors. (A)** Secreted VEGFA in the cell supernatants of SUNE1 cells and HUVECs treated with NPC exosomes, and in the cell supernatants of stable LBH overexpressing SUNE1 cells or HUVECs were measured by ELISA (***p<0.001 vs. Control; ^^^p<0.001 vs. Lv5NC). **(B)** Protein expression of LBH, p-VEGFR2, VEGFR2, p-AKT, AKT, p-P38, and P38 in tumor xenografts constructed with CNE2-Lv5NC, CNE2-LBH, SUNE1- Lv5NC, or SUNE1-LBH cells (***p<0.001 vs. Lv5NC). **(C)** Schematic summary of the LBH/CRYAB-mediated, VEGFA-VEGFR2 signaling responsible for modulating the migration/invasion and EMT process of NPC cells by autocrine signaling and for modulating the proliferation, migration and angiogenesis of HUVECs by paracrine signaling in the NPC microenvironment.

For the angiogenesis during tumor progression, the effects of VEGFA are considered to be implemented mainly via extracellular secretion. Specifically, in the tumor microenvironment, the reduced VEGFA protein levels in LBH-overexpressing NPC tissues might be due to the comprehensive effects of both NPC cells and HUVECs. Hence, we detected VEGFA levels in culture supernatants by ELISAs, and the data indicated that in both NPC cells and HUVECs, decreased intercellular VEGFA secretion could be induced by LBH+ exosome treatments or lentiviral-mediated overexpression of LBH **(Figure 8A)**. In addition, western blotting of NPC tissues showed that diminished VEGFA levels were accompanied by inhibited phosphorylation of VEGFR2, P38 and AKT, while the phosphorylation of ERK1/2 and Smad3 was unaffected **(Figure 8B, Figure S12)**. This should be interpreted as suppressed VEGFA/VEGFR2 signaling in NPC cells, since the tumor parenchyma constitutes the majority of NPC tissues, and the results are also in accordance with our discovery in HUVECs. In conclusion, elevated LBH expression inhibits the secretion of VEGFA and VEGFA/VEGFR2 signaling in NPC xenograft tumors, including in both NPC cells and HUVECs **(Figure 8C)**.

## Discussion

Intercellular communication via exosome secretion could be mediated in either autocrine or paracrine manners 39, and upregulation of cancer-associated genes in donor cells generally leads to increased levels of those genes in released exosomes 40. For NPC, exosome-mediated intercellular communication has been reported to induce relevant gene expression in recipient cells, regulating tumorigenesis and EMT, and metastasis 41, 42. In our study, the LBH protein colocalized with the vesicle marker EEA1 in NPC cells, which is consistent with its subcellular localization in vesicles (GO:0043231) approved by the Human Protein Atlas 43, implying its possible secretion via exosomes; additionally, elevated LBH at both the mRNA and protein levels was detected in exosomes derived from LBH-overexpressing NPC cells. These exosomes could be internalized by both NPC cells themselves and HUVECs, and inhibited EMT progression and angiogenic phenotypes through upregulating LBH in recipient cells. Since LBH functions as a tumor suppressor for NPC 21, LBH downregulation in NPC cells **(Figure S6A, Figure S14A)** might decrease the LBH levels in normal nasopharyngeal epithelial cells via exosome secretion in the tumor microenvironment, thereby advancing NPC progression **(Figure S14B-D)**.

VEGFA signaling has been reported to not only promote tumor angiogenesis, but to also modulate EMT progression in various tumor types 44, 45. In NPC cells treated with LBH+ exosomes, LBH upregulation was observed to synchronize with mitigated EMT progression, as well as the inhibition of CRYAB expression and phosphorylation, which is consistent with our previous results in stable LBH-overexpressing NPC cell lines 10; additionally, VEGFA expression declined upon this exosome-induced LBH upregulation. Subsequently, with introducing ectopic CRYAB expression, we verified that the LBH-mediated restraint of VEGFA signaling was partially effectuated by CRYAB downregulation, together with hampered EMT; meanwhile, ectopic VEGFA expression in LBH-overexpressing NPC cells led to enhanced EMT. These data suggest that for nasopharyngeal carcinoma, CRYAB-dependent VEGFA expression participates in regulating EMT inhibition in LBH-elevated NPC cells induced by exosome-mediated autocrine signaling, which attenuates cellular migration and invasion. This is consistent with the views of Chen et al. 46 and Schootbrugge et al. 47 that both VEGFA and CRYAB promote EMT progression and metastasis in head and neck carcinoma. The exact role of VEGFA in modulating metastasis-associated phenotypes of NPC cells affected by exosome-mediated autocrine signaling, however, requires further verification by introducing VEGFA interference during exosome treatment, for example, with receptor inhibitors, neutralizing antibodies or recombinant proteins.

For exosome-mediated paracrine signaling, NPC-derived exosomes have been reported to either enhance or suppress angiogenesis upon uptake by HUVECs due to exosomal delivery of functional biomolecules 48-50. Our Matrigel plug assay indicated that treatment with LBH+ exosomes decelerated *in vivo* angiogenesis. Additionally, HUVECs treated with LBH+ exosomes showed LBH upregulation and diminished angiogenic phenotypes, including reduced proliferation, migration and tube formation, which is in accordance with the phenotypes of LBH-overexpressing HUVECs. Moreover, similar to NPC cells, elevated LBH levels in HUVECs under the treatment of LBH+ exosomes also led to downregulated CRYAB and VEGFA, and LBH-mediated inhibition of VEGFA signaling was also mediated by CRYAB. These findings agree with the findings of Conen et al. that LBH negatively regulates VEGFA signaling and angiogenesis 51, while CRYAB positively regulates these processes 52, 53. Hence, we concluded that in NPC tumors, LBH-mediated inactivation of CRYAB and VEGFA in HUVECs can be induced by LBH+ NPC exosome in a paracrine manner, thus impairing NPC tumor angiogenesis.

CRYAB's regulation of VEGFA signaling has been reported to be achieved through PPIs; the interaction between phosphorylated CRYAB (Ser59) and misfolded VEGFA protein obviated VEGFA degradation and resulted in escalated VEGFA secretion 17, 38, 52. Specifically for NPC, the work by Schootbrugge et al. indicates that CRYAB stimulates VEGFA secretion, tumor cell migration and correlates with enhanced distant metastasis 54. Therefore, the potential mechanisms by which LBH restrains VEGFA signaling via CRYAB downregulation need further investigation. Since CRYAB has been identified as a scaffold to complex with other proteins for signal transduction 55, 56 and the PPIs between LBH and CRYAB in NPC cells were confirmed in our previous research 10, we tested the potential PPIs between CRYAB and VEGFA, as well as between LBH and VEGFA in HUVECs. Significant FRET effects verified that VEGFA protein interacts with both LBH and CRYAB proteins in HUVECs. Based on these results, we hypothesized that the CRYAB-dependent LBH regulation of VEGFA signaling might be due to PPIs among these proteins, in which CRYAB interacts with both LBH and VEGFA, tethering them into functional complexes. However, this VEGFA regulation does not necessarily rely on PPIs; in fact, CRYAB has been reported to be transcriptionally regulated under exogenous TGF-β1 stimulation 57, while LBH has been verified to function as a transcriptional cofactor in NPC, and to be regulated by TGF-β1 signaling as a downstream factor 21. Additionally, we demonstrated that CRYAB might be transcriptionally regulated by both TGF-β1 and LBH in our previous studies 10, 58. Since there is no external evidence to support that VEGFA is transcriptionally regulated by LBH as we observed, it could be our next research orientation to further explore the potential mechanisms.

It is generally accepted that the effectuation of VEGFA signaling relies on the binding of extracellular secreted VEGFA protein with its membrane receptors to activate receptor phosphorylation and subsequent pathways. VEGFA_165_ and VEGF receptor-2 (VEGFR2) have been identified as the predominant isoforms for VEGFA signaling in HUVECs 59, and the angiogenic downstream cascades of VEGFA-VEGFR2 signaling have been investigated intensively 60, in which the phosphorylation of AKT, ERK, p38 and Smad 2/3 has been discussed most frequently. In this study, we checked these downstream cascades, and demonstrated that the suppressed VEGFA expression and VEGFR2 phosphorylation mediated by LBH+ exosomes inhibited the phosphorylation of AKT and p38 in HUVECs, whose activation correspond to attenuated proliferation and migration of HUVECs, respectively 61, 62. The phosphorylation of ERK 1/2 and Smad 2/3, however, was unaffected. Meanwhile, the inhibited phosphorylation of AKT and p38 detected in LBH-overexpressing xenograft tissues should be interpreted as being correlated with declined CRYAB and VEGFA levels in LBH-overexpressing NPC cells. Whereas, studies of VEGFA-VEGFR signaling in tumor cells have reached controversial conclusions (including the idea that p38/AKT phosphorylation regulates VEGFA expression; VEGFA regulates p38/AKT phosphorylation; VEGFA and p38/AKT phosphorylation mutually activate each other through a feedback loop) 59, 63-65. Thus, the exact relationships among VEGFA, AKT and p38 in NPC cells merit further exploration. Moreover, VEGFA downregulation led to less extracellular VEGFA secretion from both NPC cells and HUVECs treated with LBH+ exosomes, resulting in decreased VEGFA levels in the interstitium of NPC tissues and weakened phosphorylation of VEGFR, which is in accordance with the mitigated EMT progression and angiogenesis we observed. The functions of VEGFA in binding and activating intracellular VEGFR, which has been defined as intracrine by some researchers 66, 67, were not under our consideration due to a lack of convincing evidence.

Anti-angiogenesis is considered a crucial adjuvant treatment with radiation therapy, which is currently the first line treatment for NPC 68. VEGFA has been identified as a therapeutic target of tumor angiogenesis for decades 69 and has been reported to be associated with promoted EMT progression in various cancer types. Based on our research, we postulate that specifically upregulating LBH during NPC therapy might ameliorate tumor metastasis and angiogenesis, achieving better therapeutic efficacy for NPC patients via exosome-mediated VEGFA inhibition, and this is likely our next research topic.

## Conclusion

Collectively, this study explored the mechanisms by which exosome-derived LBH modulates the progression of nasopharyngeal carcinoma (**Figure 8 C**). LBH protein in NPC cells can be transferred into HUVECs and themselves via paracrine and autocrine signaling mediated by exosomes; elevated LBH levels in recipient cells inhibit CRYAB-dependent VEGFA expression and secretion, which might be mediated by protein-protein interactions; downregulated VEGFA-VEGFR signaling alleviates EMT progression and angiogenesis in NPC. Our findings indicate that LBH could serve as a potential research or therapeutic target in VEGFA-focused NPC treatment.

## Supplementary Material

Supplementary figures and tables.
