# Supplementary material for: Exosomal LBH inhibits epithelial-mesenchymal transition and angiogenesis in nasopharyngeal carcinoma via downregulating VEGFA signaling
Source: Int J Biol Sci. 2022 Jan 1;18(1):242–60. doi: 10.7150/ijbs.66506 (PMC8692147; doi:10.7150/ijbs.66506)
Supplement: Supplementary file 1 — Supplementary figures and tables. [file ijbsv18p0242s1.pdf]

# Supplemental Tables and Figures

## Supplemental Table 1. Primer sequences for real-time PCR

| **Gene** | Forward (5' to 3') | Reverse (5' to 3') |
|---|---|---|
| human LBH | ctctgactatctgagatcggctgag | gcaggcggtccttcagtttg |
| human VEGFA | cagaatcatcacgaagtggtg | gaagatgtccaccagggtc |
| human CRYAB | agagcacctgttggagtctgatc | ggagaagtgcttcacatccaggt |
| human Vimentin | cgtttccaagcctgacctcac | gcatccacttcgcaggtgag |
| human E-cadherin | cacctgattcttaggcagatgcca | gtggtcaggttcactggcatg |
| human GAPDH | ggcaaggtcatcccagagct | cccaggatgccctttagtgg |
| human CD63 | cagtggtcatcatcgcagtg | atcgaagcagtgtggttgttt |

GAPDH, glyceraldehyde-3-phosphate dehydrogenase; CRYAB, αB-Crystallin.

## Supplemental Table 2. Antibodies for western blotting and Immunofluorescence

| **Antibodies** | **Product numbers** | **Applications** |
|---|---|---|
| rabbit anti-LBH | Abcam, ab173737 | WB (1:100) |
| rabbit anti-LBH | Abcam, ab122223 | IF (1:100) |
| rabbit anti-CRYAB | Abcam, ab76467 | WB (1:500) |
| rabbit anti-phospho-CRYAB | Abcam, ab5577 | WB (1:2000); IF (1:100) |
| mouse anti-EEA1 | Abcam, ab70521 | IF (1:100) |
| mouse anti-Vimentin | Boster, BM0135 | WB (1:400) |

| | | |
|---|---|---|
| mouse anti-E-cadherin | BD, 610182 | WB (1:2000) |
| mouse anti-Snail | CST, 3895S | WB (1:800) |
| rabbit anti-Slug | Boster, PB0443 | WB (1:400)<br>WB (1:800) |
| | CST,46702S | WB (1:800); |
| rabbit anti-Twist I | Proteintech, 66828-1 | IF/IHF (1:100) |
| mouse anti-VEGFA | CST, 2478S | WB/ICW |
| rabbit anti-phospho- | | (1:800) |
| VEGFR2 | Proteintech,67407-1 | WB/ICW |
| rabbit anti-VEGFR2 | Proteintech, 27309-1 | (1:500) |
| rabbit anti-Ki67 | CST, 9520S | WB (1:800); IF |
| rabbit anti-phospho-Smad3 | | (1:100) |
| rabbit anti-Smad3 | CST, 9523S | WB (1:800) |
| rabbit anti-phospho-ERK- | CST, 9106S | WB (1:800) |
| 1/2 | | WB (1:800) |
| rabbit anti- ERK-1/2 | CST, 9102S | WB (1:800) |
| rabbit anti-phospho-AKT | CST, 4060S | WB (1:800) |
| rabbit anti-AKT | | |
| | CST, 9272S | WB (1:800) |
| rabbit anti-p38 | CST, 9212S | WB (1:800) |
| rabbit anti-phospho-p38 | CST, 9211S | WB (1:800) |
| rabbit anti-GAPDH | Bioworld, AP0063 | WB (1:8000) |

| | | |
|---|---|---|
| mouse anti-α-Tubulin | Proteintech, 66031-1 | WB (1:2000) |
| rabbit anti-CD31 | Abcam, ab24590 | IF (1:100) |
| rabbit anti-CD34 | Abcam, ab81289 | IF/IHF (1:100) |
| rabbit anti-CD9 | Bioss, bs-2489R | WB (1:500) |
| rabbit anti-CD63 | Abcam, ab134045 | WB (1:1000) |
| rabbit anti-CD81 | Bioss, bs-6934R | WB (1:500) |
| goat anti-rabbit IgG-HRP | CST, 7074 | WB (1:4000) |
| horse anti-mouse IgG-HRP | CST, 7076 | WB (1:4000) |
| goat anti-mouse-Alexa Fluor 488 | Invitrogen A-11001 | IF (1:500) |
| goat anti-mouse-Alexa Fluor Plus 555 | Invitrogen A-21422 | IF (1:500) |
| goat anti-mouse-Alexa Fluor Plus 633 | Invitrogen A-21052 | IF (1:500) |
| donkey anti-rabbit-Alexa Fluor Plus 555 | Invitrogen A-31572 | IF/IHF (1:500) |
| goat anti-mouse Alexa Fluor Plus 680 | Invitrogen A-21057 | IHF/ICW (1:500) |
| goat anti-rabbit Alexa Fluor Plus 790 | Invitrogen A-11369 | IHF/ICW (1:500) |
| rabbit-anti-LBH-FTIC | Lifespan LS-C672608 | NFC (1:25) |

WB, Western blotting; IF, Immunofluorescence; IHF, Immunohistofluorescence; ICW, In cell western; NFC, Nano-flow cytometry.

## Supplemental Figure 1

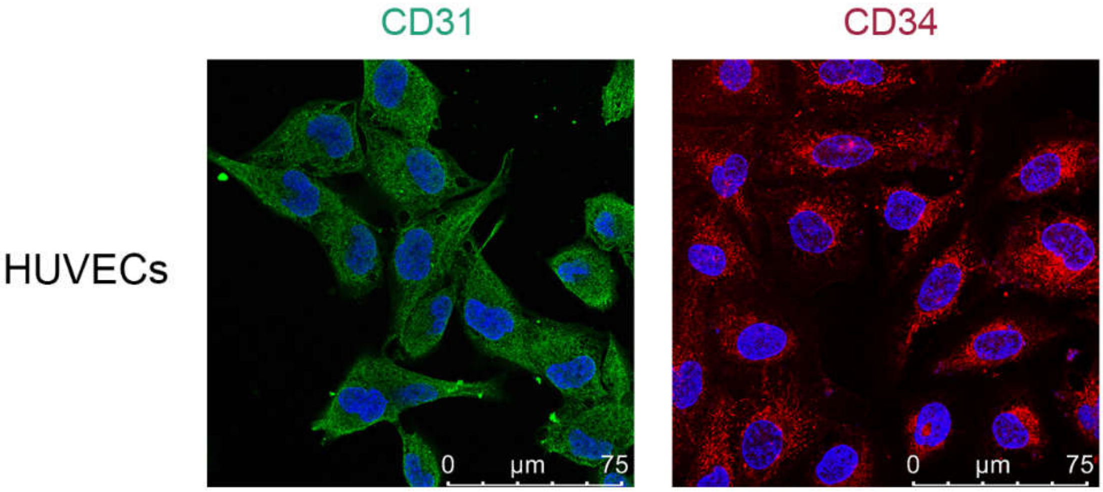


**Supplemental Fig. 1** Representative immunofluorescence images of HUVECs for the staining of CD31 and CD34. The fact that the majority of tested cells were $CD31^{+}$ and $CD34^{+}$ verified the purity of HUVECs.

## Supplemental Figure 2

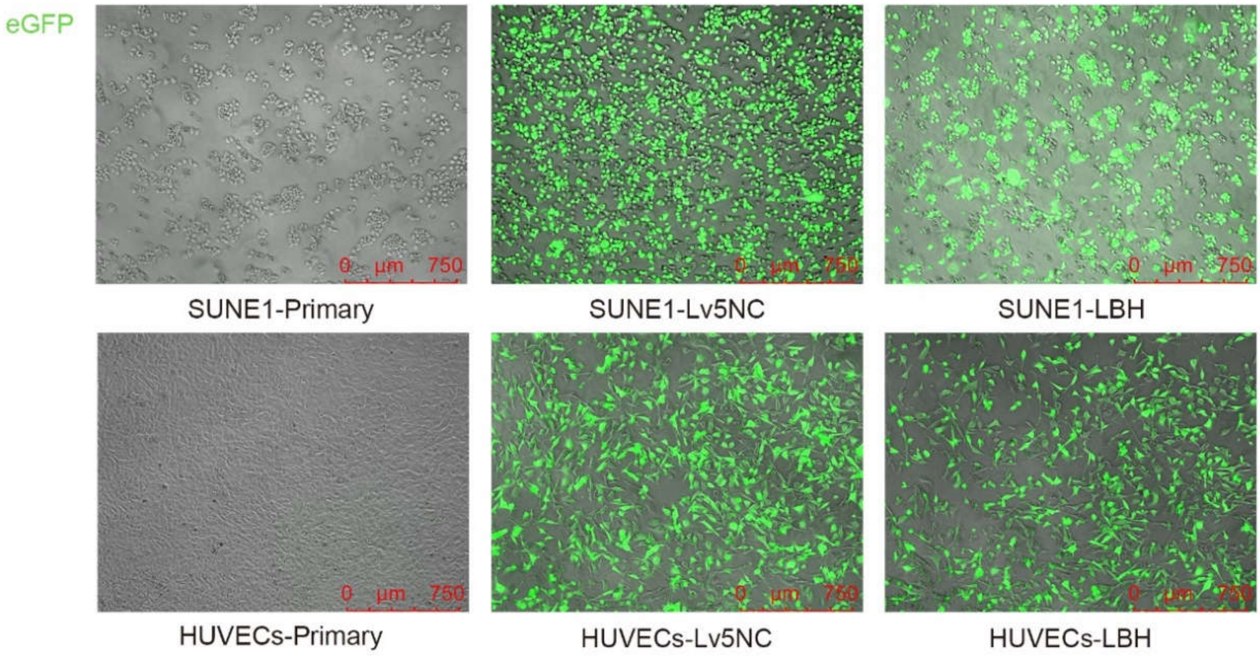


**Supplemental Fig. 2** Fluorescence images indicating the infection efficiencies of SUNE1 and HUVECs cell lines prepared for the following experiments. The green fluorescence is expressed eGFP which was constructed in Lentivirus vectors.

## Supplemental Figure 3

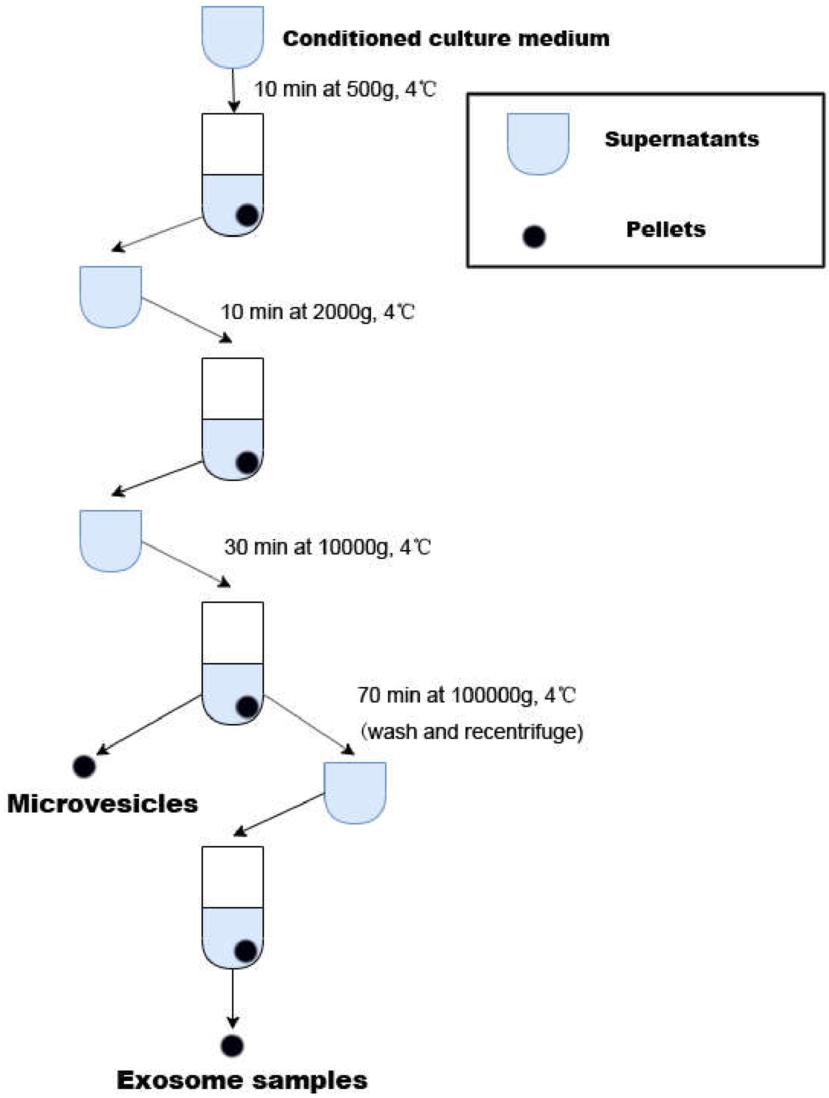


**Supplemental Fig. 3** Protocols of exosome isolation by differential ultracentrifugation used in this study, presented as flowchart.

## Supplemental Figure 4

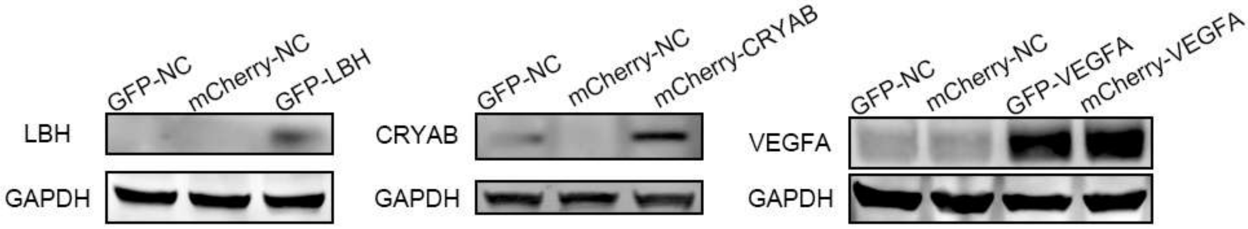

**Supplemental Fig. 4** Representative image of Western blotting performed to validate the exogenous expressions of designated genes initiated by transfecting reconstructed plasmids into HUVECs. It confirmed the functions of reconstructed plasmids used for FRET assays in **Fig.7B.**

## Supplemental Figure 5

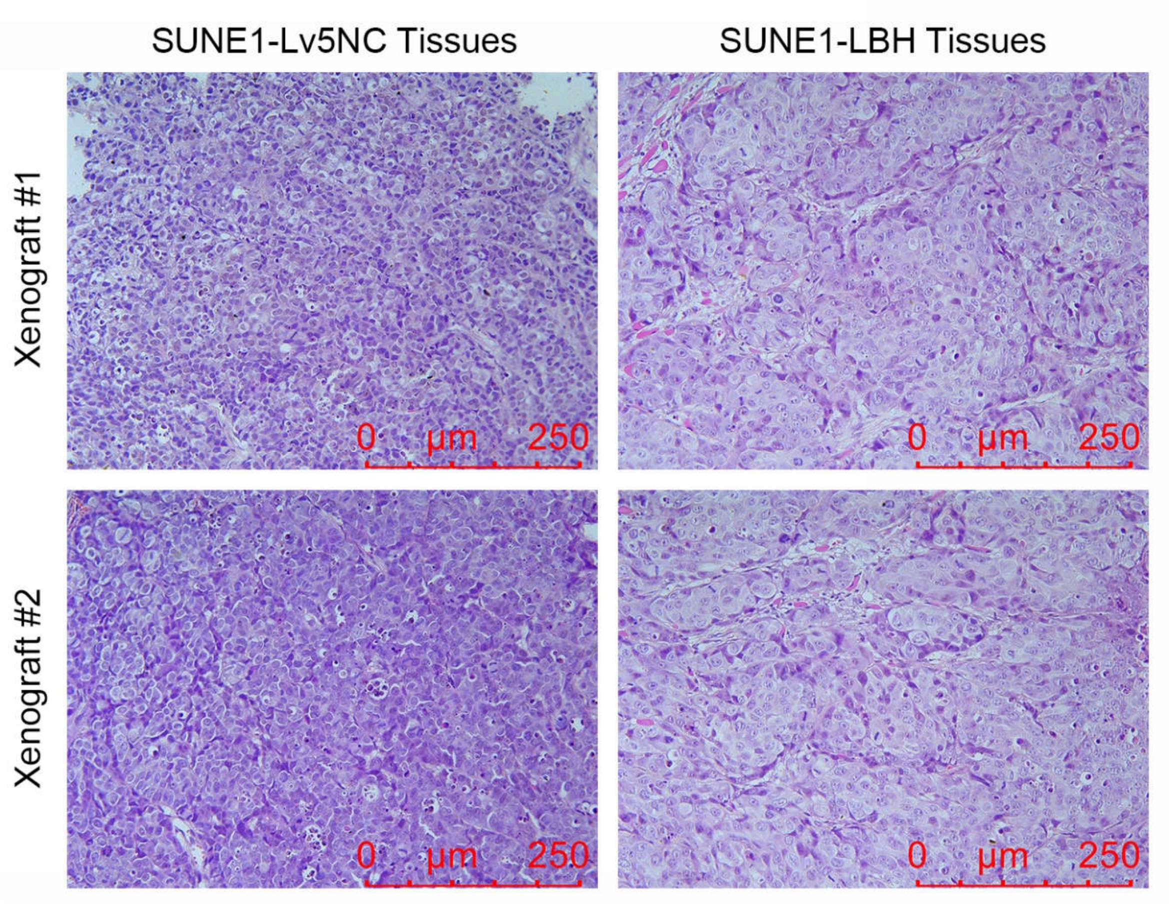


**Supplemental Fig. 5** H&E staining of tumor xenografts constructed by LBH-overexpressed NPC cells. For SUNE1 NPC xenografts, the tissues of LBH-overexpressed tumors are well differentiated compared to the controls, which exhibited epithelial characteristics, while the tissues of Lv5NC tumors exhibited poorly differentiated, mesenchymal characteristics.

## Supplemental Figure 6

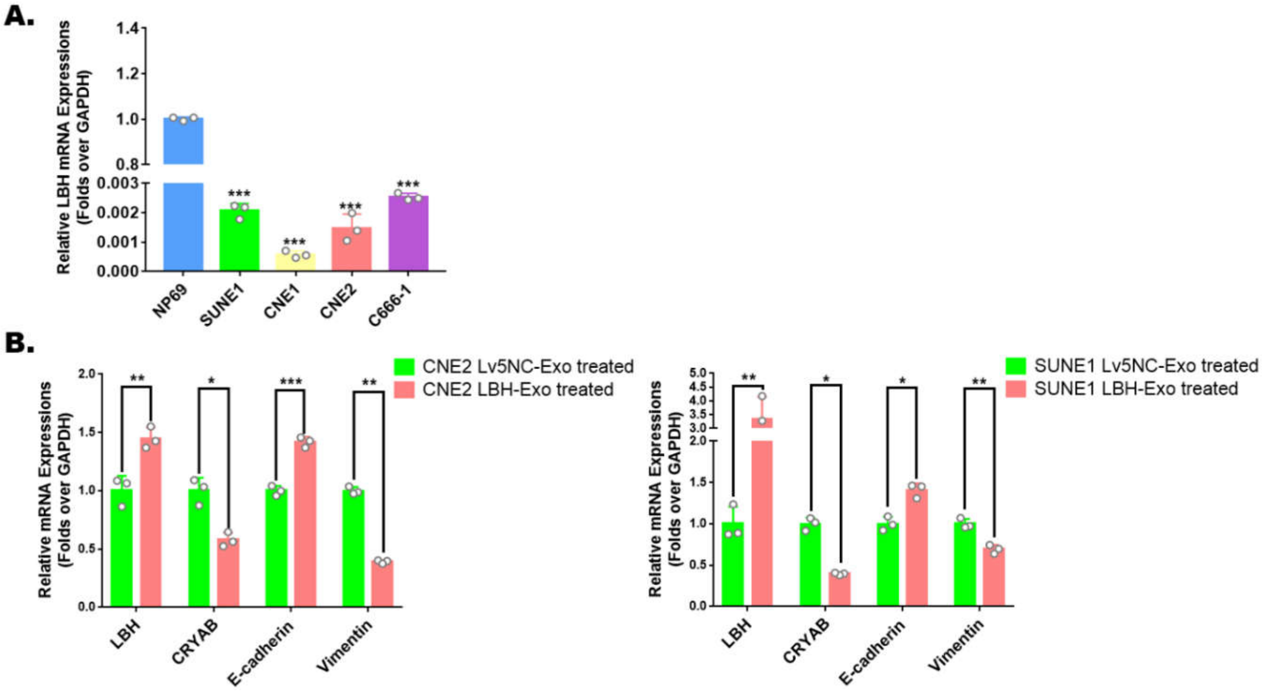


**Supplemental Fig. 6 (A)** mRNA levels of LBH in NP69 and NPC cell lines (***p<0.001 vs. NP69). **(B)** mRNA levels of LBH, CRYAB, E-cadherin and Vimentin in NPC cells treated by NPC exosomes (*p<0.05, **p<0.01 and ***p<0.001 vs. Lv5NC-Exo treated).

## Supplemental Figure 7

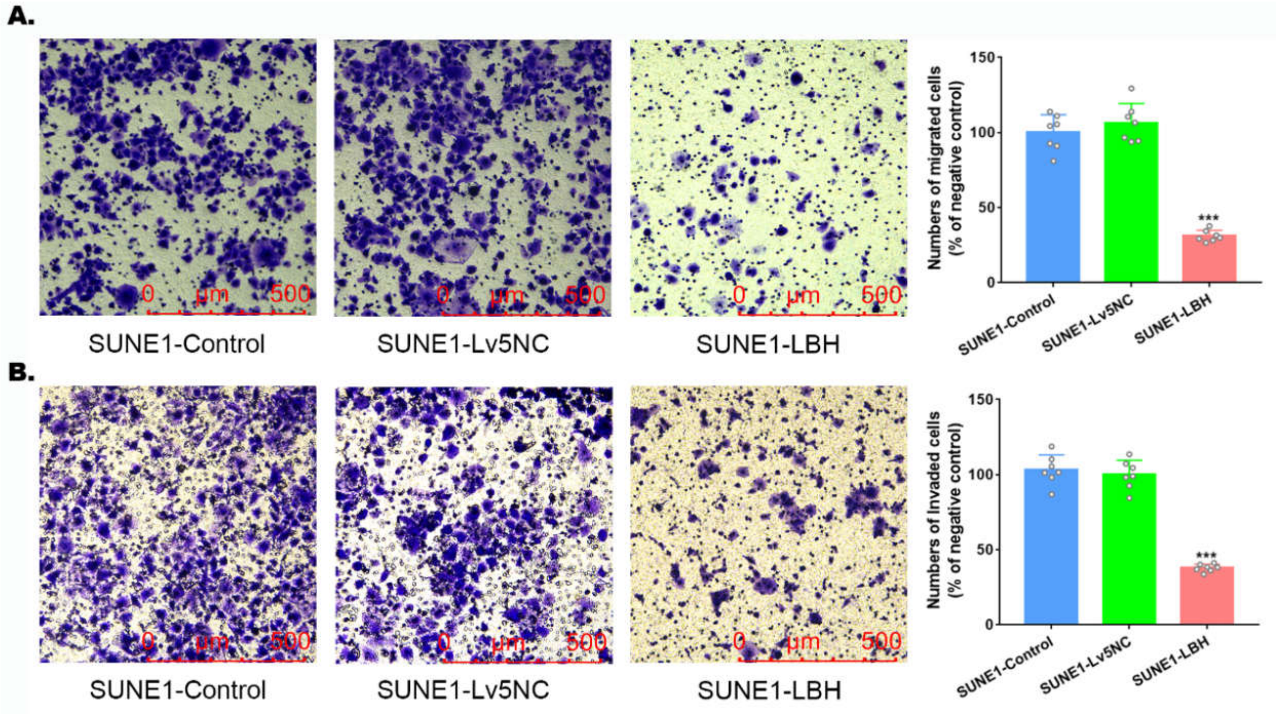

**Supplemental Fig. 7** Representative images of the Transwell assay **(A)** and Matrigel Transwell assay **(B)** of SUNE1-Control, SUNE1-Lv5NC and SUNE1-LBH, and corresponding statistical analyses (***p<0.001 vs. Control).

## Supplemental Figure 8

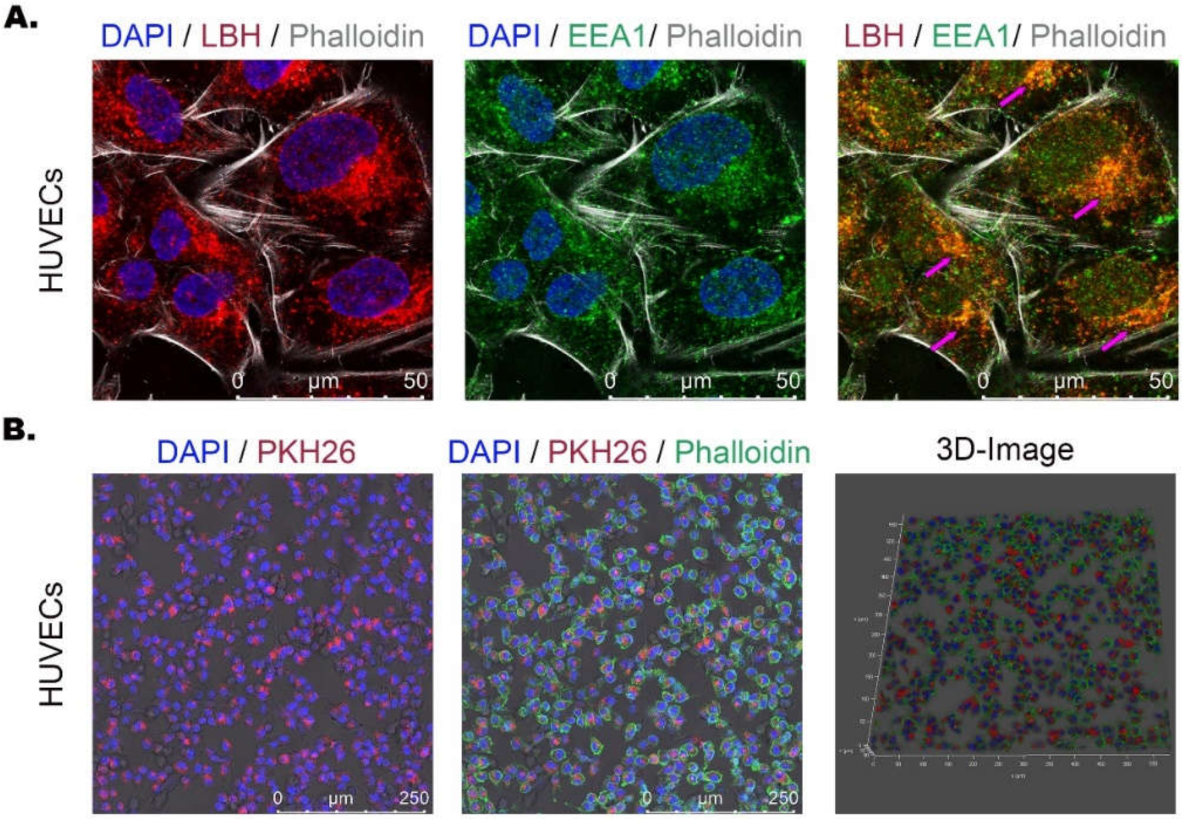


**Supplemental Fig. 8 (A)** Representative confocal microscopic images of dual staining of anti-LBH (red) and anti-EEA1 (green) in HUVECs. Colocalization of LBH and EEA1 were observed in the perinuclear cytoplasm of HUVECs (Indicated by magenta arrows). **(B)** Representative confocal microscopic images of HUVECs treated by PKH26 labelled exosomes derived from SUNE1 cells. Both the 2D fluorescence images and the 3D reconstructed image confirmed the intracellular distribution of PKH26 labelled exosomes inside HUVECs, and some cells in the DAPI/PKH26/Phalloidin channel showed ongoing membrane fusion, which was indicated by merged signal of PKH26 and Phalloidin.

## Supplemental Figure 9

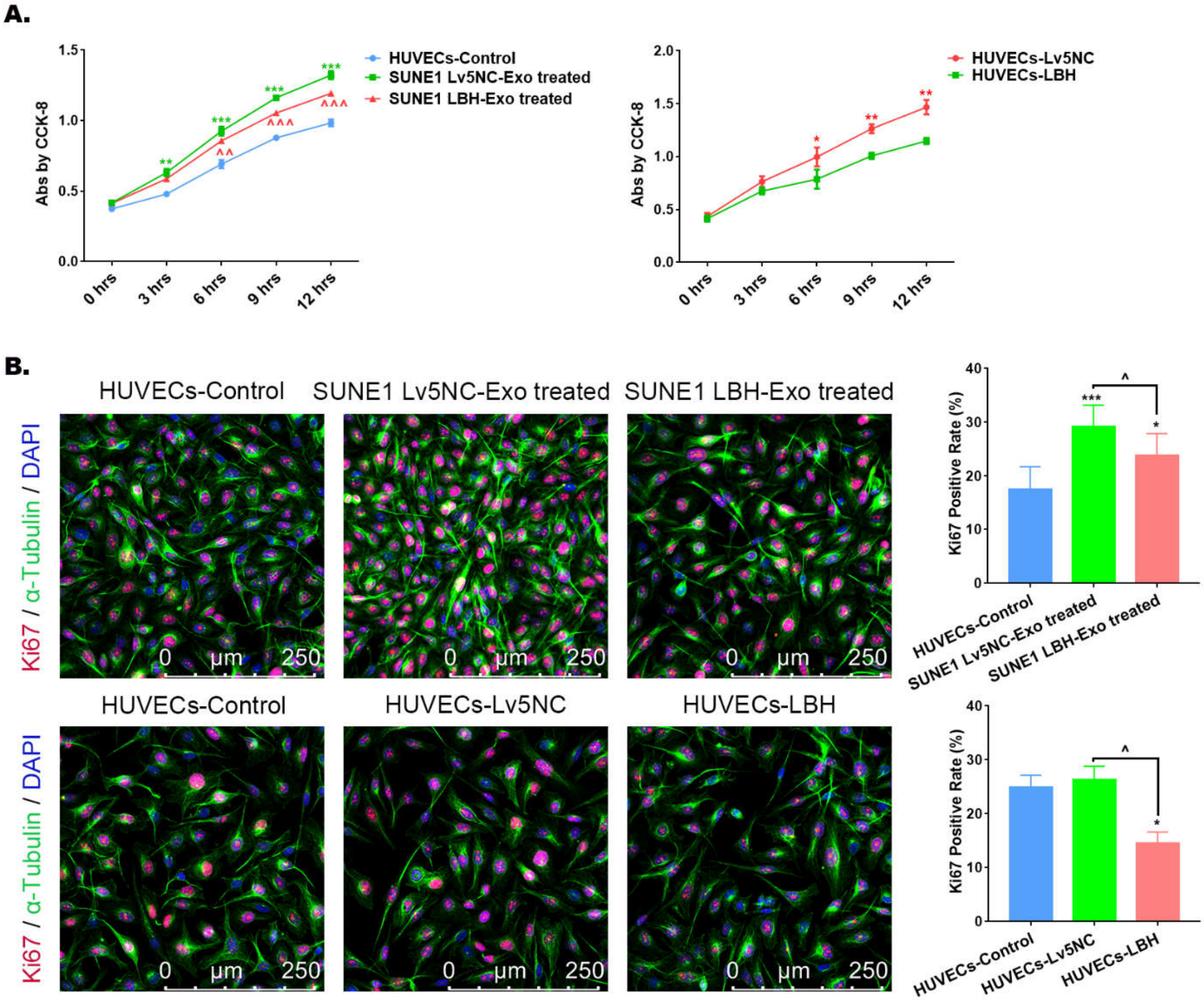


**Supplemental Fig. 9** Cellular proliferation for HUVECs infected with LBH-overexpressed lentivirus or treated with NPC exosomes from LBH-overexpressed SUNE1 cells. Both the CCK-8 data and immuno-staining of Ki67 are in agreement with its proliferation of the same samples detected by EdU staining in **Fig. 5A**.

## Supplemental Figure 10

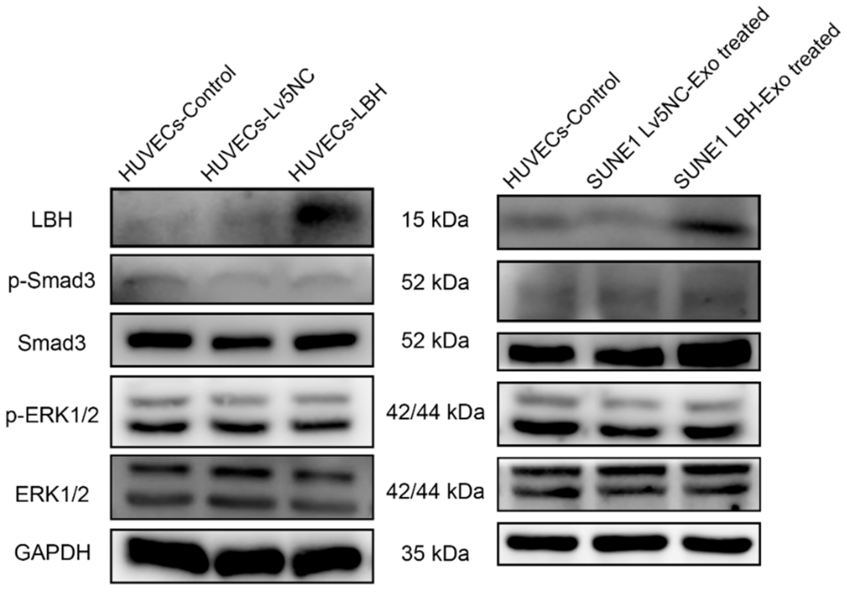


**Supplemental Fig. 10** Part of downstream cascades in VEGFA/VEGFR2 signaling in HUVECs that was not affected by LBH-overexpression or NPC exosomes treatment. The WB results of the parallel cascades of VEGFA/VEGFR2 signaling, namely AKT and P38 pathways were showed in **Fig. 6B/C**.

## Supplemental Figure 11

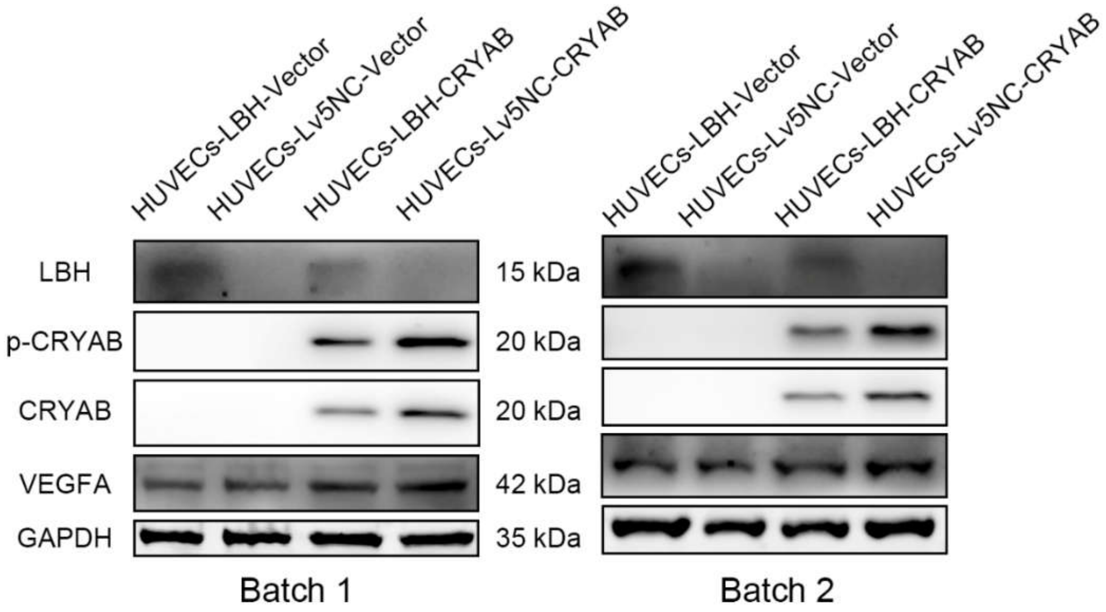


**Supplemental Fig. 11** Representative image of Western blotting detecting HUVECs-Lv5NC and HUVECs-LBH transfected with mCherry-CRYAB plasmid. The fact that CRYAB overexpression reversed the VEGFA inhibition mediated by LBH

upregulation confirmed that the function of LBH to inhibit VEGFA signaling is modulated by downregulating CRYAB in HUVECs.

## Supplemental Figure 12

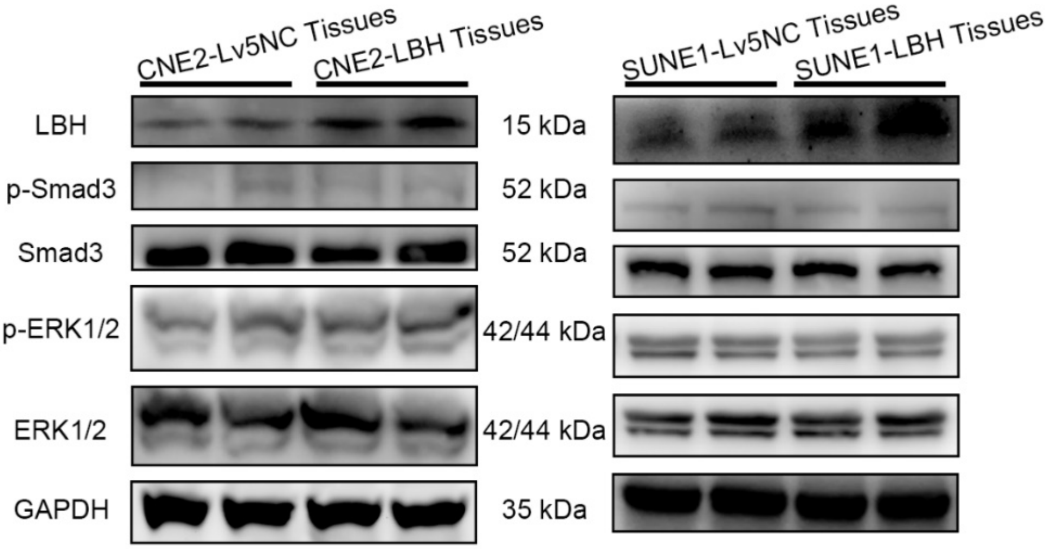


**Supplemental Fig. 12** Part of downstream cascades in VEGFA/VEGFR2 signaling in NPC xenograft tumor tissues that was not affected by LBH-overexpression. The WB results of the parallel cascades of VEGFA/VEGFR2 signaling, namely AKT and P38 pathways were showed in **Fig. 8B**.

## Supplemental Figure 13

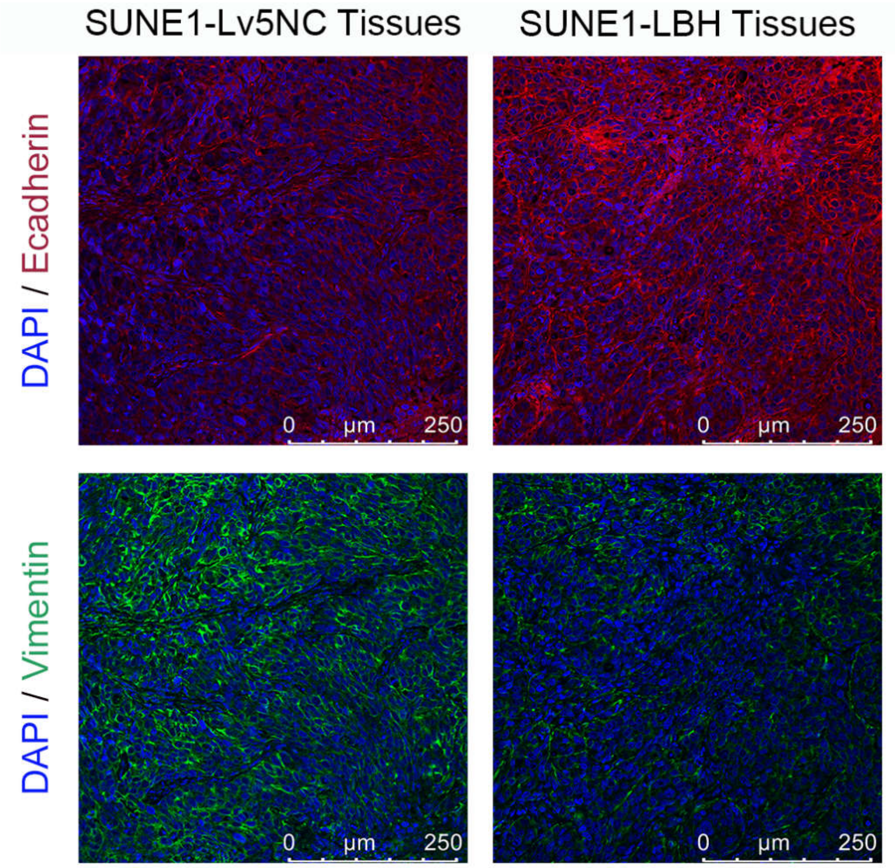


**Supplemental Fig. 13** EMT progressions of tumor xenografts constructed by LBH-overexpressed NPC cells were indicated by co-staining of anti-E-cadherin and anti-Vimentin. For SUNE1 NPC xenografts, the tissues of LBH-overexpressed tumors exhibited upregulated epithelial marker E-cadherin, as well as downregulated mesenchymal marker Vimentin compared to the tissues of Lv5NC tumors, which implied that LBH overexpression is correlated with inhibited EMT of NPC tumor xenografts.

## Supplemental Figure 14

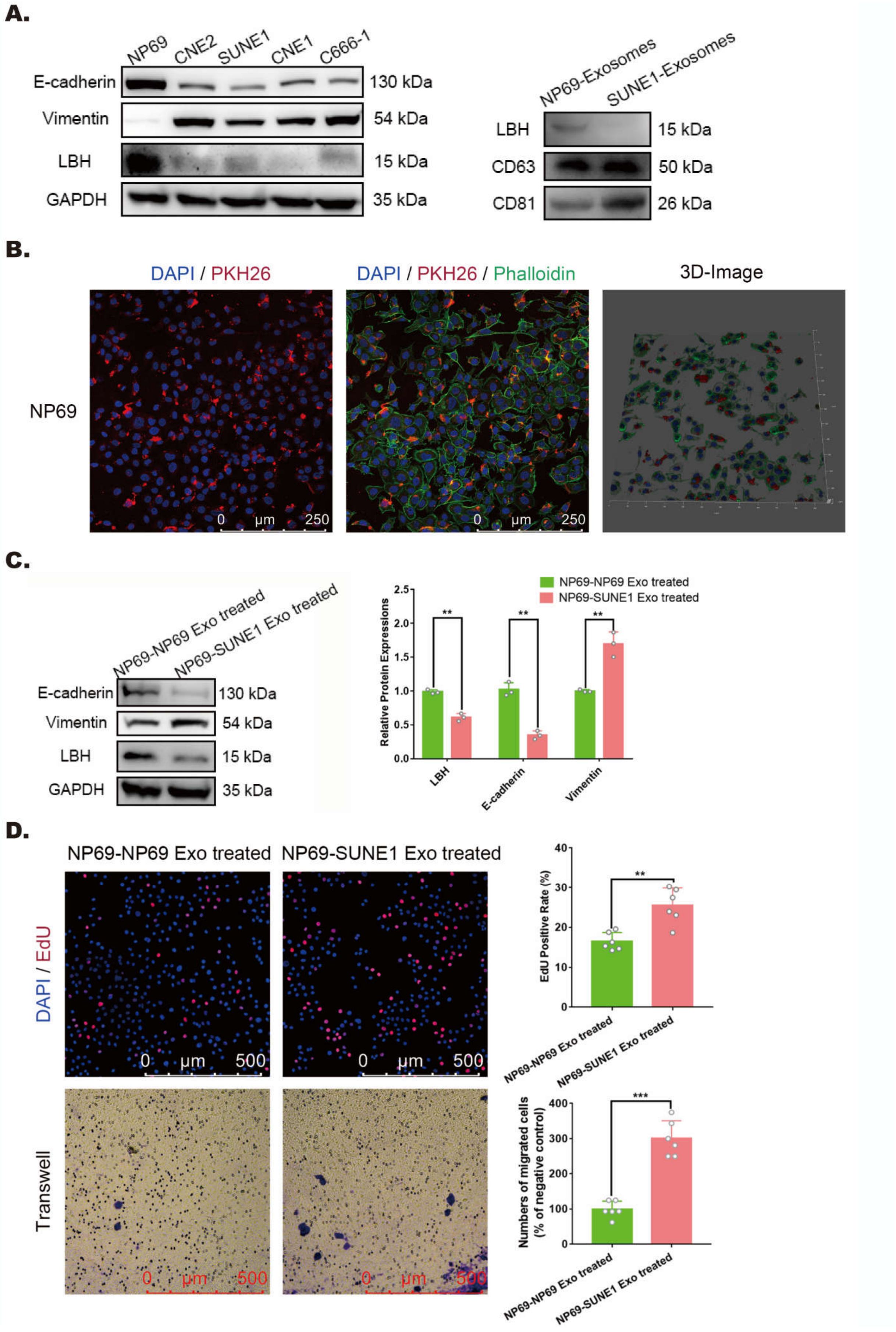

A.
NP69
CNE2
SUNE1
CNE1
C666-1
E-cadherin
Vimentin
LBH
GAPDH
130 kDa
54 kDa
15 kDa
35 kDa
NP69-Exosomes
SUNE1-Exosomes
LBH
CD63
CD81
15 kDa
50 kDa
26 kDa
B.
DAPI / PKH26
DAPI / PKH26 / Phalloidin
3D-Image
NP69
0 μm 250
0 μm 250
C.
NP69-NP69 Exo treated
NP69-SUNE1 Exo treated
E-cadherin
Vimentin
LBH
GAPDH
130 kDa
54 kDa
15 kDa
35 kDa
Relative Protein Expressions
LBH
E-cadherin
Vimentin
D.
NP69-NP69 Exo treated
NP69-SUNE1 Exo treated
DAPI / EdU
Transwell
0 μm 500
EdU Positive Rate (%)
Numbers of migrated cells (% of negative control)

**Supplemental Fig. 14** The effects of LBH+ NPC exosomes on nasopharyngeal epithelial cell line NP69. **(A)** Western blotting verified elevated LBH expression and epithelial characteristics in NP69 cells compared to multiple NPC cell lines; NP69-derived exosomes showed higher LBH protein level compared to NPC-derived exosomes. **(B)** Representative confocal microscopic images of NP69 treated by PKH26 labelled exosomes derived from SUNE1 cells. Both the 2D fluorescence images and the 3D reconstructed image confirmed the intracellular distribution of PKH26 labelled exosomes inside NP69 cells. **(C)** Protein levels of LBH, Vimentin and E-cadherin in NP69 cells treated with NP69-derived exosomes or SUNE1-derived exosomes (**p<0.01 vs. Lv5NC-Exo treated). **(D)** Representative images of the Transwell assay and EdU assay of NP69 cells treated with NP69-derived exosomes or SUNE1-derived exosomes, and corresponding statistical analysis (**p<0.01 and ***p<0.001 vs. Lv5NC-Exo treated).
